# Unravelling the electron injection/transport mechanism in organic light-emitting diodes

**DOI:** 10.1038/s41467-021-23067-2

**Published:** 2021-05-11

**Authors:** Tsubasa Sasaki, Munehiro Hasegawa, Kaito Inagaki, Hirokazu Ito, Kazuma Suzuki, Taku Oono, Katsuyuki Morii, Takahisa Shimizu, Hirohiko Fukagawa

**Affiliations:** 1 Japan Broadcasting Corporation (NHK), Science & Technology Research Laboratories, Setagaya-ku, Tokyo Japan; 2 Nippon Shokubai Co., Ltd., Suita, Osaka Japan; 3 Department of Physics, Graduate School of Science, Tokyo University of Science, Tokyo, Japan; 4 Nippon Shokubai Research Alliance Laboratories, Osaka University, Osaka, Japan

**Keywords:** Electronic devices, Organic LEDs, Electronic structure of atoms and molecules

## Abstract

Although significant progress has been made in the development of light-emitting materials for organic light-emitting diodes along with the elucidation of emission mechanisms, the electron injection/transport mechanism remains unclear, and the materials used for electron injection/transport have been basically unchanged for more than 20 years. Here, we unravelled the electron injection/transport mechanism by tuning the work function near the cathode to about 2.0 eV using a superbase. This extremely low-work function cathode allows direct electron injection into various materials, and it was found that organic materials can transport electrons independently of their molecular structure. On the basis of these findings, we have realised a simply structured blue organic light-emitting diode with an operational lifetime of more than 1,000,000 hours. Unravelling the electron injection/transport mechanism, as reported in this paper, not only greatly increases the choice of materials to be used for devices, but also allows simple device structures.

## Abstract (web summary)

Understanding the role electron injection and transport in organic light-emitting diodes (OLED) is critical for optimizing device performance. Here, the authors elucidate the electron injection/transport mechanism in OLEDs and identify the cathode/emissive layer energy barrier as the key factor.

## Introduction

Starting with the report by Tang and VanSlyke in 1987, organic light-emitting diodes (OLEDs) have been studied intensively, and their practical application is the most advanced among organic devices^1^. Over the past 30 years, remarkable progress has been made in improving the efficiency of light-emitting materials through, for example, the understanding of the triplet–triplet annihilation (TTA) in fluorescent OLEDs and the discovery of both phosphorescent emitters and thermally activated delayed fluorescent (TADF) emitters^2–6^. On the other hand, the materials used between the cathode and the emitting layer (EML), which includes the light-emitting material, have remained basically unchanged^7^. Since the electron affinity (EA) of the materials used in OLEDs is smaller than that of the materials used in other organic devices, chemically reactive alkali elements such as Li/Cs or molecular n-dopants have been essential for the electron injection layer (EIL) near the cathode^7–10^. In addition, compounds with nitrogen-containing heterocycles, such as pyridine, imidazole and phenanthroline, have been typically defined as electron transport materials (ETMs), and intensive efforts have been made on developing ETMs with high electron mobility^11–19^. The use of both EIL and typical ETMs is now almost seen as “conventional wisdom” that has been gained through the development of numerous OLEDs; however, the detailed mechanisms involved in electron injection/transport from the cathode to the EML have remained unclear, such as whether injection or transport is more important to operate OLEDs at a low operating voltage^7–20^. Since n-dopants change both injection and transport properties, it has been difficult to distinguish their effects on the operating voltage^7–10,12^. In the development of ETMs used for OLEDs, the operating voltage had mainly been discussed on the basis of the electron mobility of ETMs, whereas the effect of the electron injection property on the operating voltage has rarely been discussed^11–19^. That is because little has been known about the actual EA of organic compounds with a small EA until recently^21,22^.

Here, we elucidated the electron injection/transport mechanism in OLEDs by correlating the characteristics of OLEDs with the actual energy levels around cathodes. The only requirement to obtain OLED emission at a low operating voltage was found to be the minimisation of the energy barrier between the cathode and the EML. Compounds with nitrogen-containing heterocycles, which have been considered as typical ETMs, are found to perform electron injection rather than electron transport. We demonstrate that these typical ETMs are not essential for OLEDs by finding an EIL that can significantly reduce the work function (WF) around the cathode. The realisation of a low-WF electrode to eliminate reactive alkali elements from organic devices has been the subject of intense study^9,23–27^. Zhou et al. reported various low-WF electrodes with a minimum WF of 3.1 eV by utilising polyethyleneimine^23^. Lin et al. have fabricated an organic film with a surface WF of about 2.5 eV by photoactivation of a molecular n-dopant doped into a typical ETM^9^. Very recently, Tang et al. have realised a low effective WF of 2.4 eV using multivalent anions^24^. Both Bin et al. and the present authors reported some phenanthroline derivatives that can reduce the WF of electrodes to below 3 eV by utilising the coordination reaction from phenanthroline derivatives with metals^25,26^. We also demonstrated that the formation of hydrogen bonds (H-bonds) between nitrogen in bases and other organic semiconductors reduces the WF to about 3 eV^27^. The EIL found in this study is the superbase 2,6-bis(1,3,4,6,7,8-tetrahydro-2H-pyrimido[1,2-a]pyrimidin-1-yl)pyridine (Py-hpp_2_), which can reduce the WF near an Al cathode to about 2.0 eV through both the coordination reaction and the formation of H-bonds^28^. This extremely low WF allows direct electron injection into materials used in blue and green EMLs with a relatively large band gap. The luminance of blue OLEDs without typical ETMs was increased by about 200,000 times at an applied voltage of 6 V by changing the EIL from a Li compound to the EIL.

## Results

### Effect of electron injection barrier on operating voltage

**Fig. 1:**
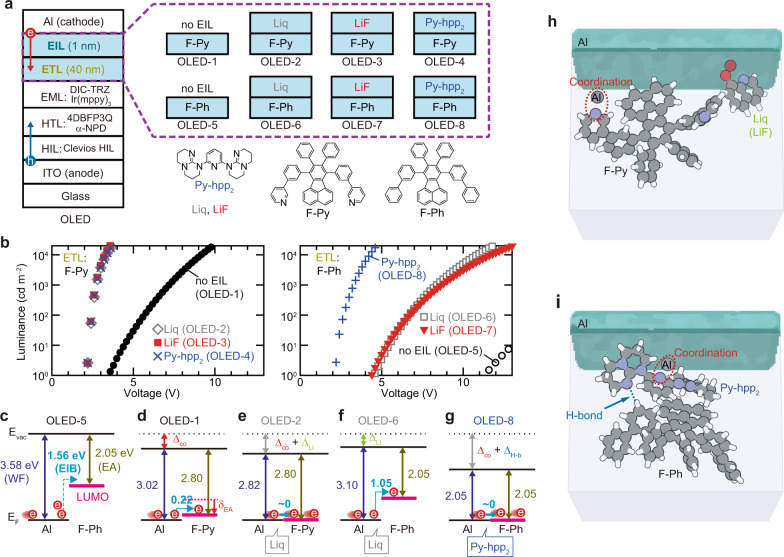
Characteristics of OLEDs with different EIL/ETL combinations. **a** Multilayer structure of an OLED, chemical structure of the materials used in the ETL and EIL, and schematic illustrations of their combination. **b** Luminance–voltage characteristics of green phosphorescent OLEDs prepared using various EIL/ETL combinations. **c**–**g** Summary of the energy diagrams around each cathode estimated from UPS and LEIPS, where E_F_ and E_vac_ represent the Fermi and vacuum levels, respectively. **h**, **i** Schematic illustrations of nitrogen-induced interactions around the cathode such as the coordination reaction with diffused Al atoms and the formation of H-bonds between Py-hpp_2_ and the material used in the ETL.

The electron injection/transport mechanism was clarified by investigating the EIL- and electron transporting layer (ETL)-dependent characteristics of the green phosphorescent OLEDs shown in Fig. 1a and b (Supplementary Fig. 1). In addition to typical EILs such as lithium fluoride (LiF) and (8-quinolinolato)lithium (Liq), Py-hpp_2_ was also used^28^. Two similar materials were used for the ETL shown in Fig. 1a: one was 7,10-bis(3-(pyridin-3-yl)phenyl)-8,9-diphenylfluoranthene (F-Py), which is considered to be a typical ETM owing to the existence of the pyridine substituent, and the other was 7,10-bis(biphenyl-3-yl)-8,9-diphenylfluoranthene (F-Ph). There are no significant differences in EA measured by low-energy inverse photoemission spectroscopy (LEIPS) and the lowest unoccupied molecular orbital (LUMO) between F-Ph and F-Py (Supplementary Fig. 2)^21,22^. However, both the turn-on voltage at 1 cd m^–2^ and the operating voltage were found to be highly dependent on the material used in the ETL for the OLEDs with no EIL (OLED-1 and OLED-5) and for the OLEDs with Li compounds (OLED-2, OLED-3, OLED-6 and OLED-7), as shown in Fig. 1b. According to conventional wisdom, these results can be attributed to the electron transportability of F-Py being higher than that of F-Ph. Note the difference in turn-on voltage between OLED-5 with no EIL and OLED-8 with Py-hpp_2_, which is as high as about 10 V. In addition, the turn-on voltage is almost independent of the material used in the ETL when Py-hpp_2_ was used for the EIL (OLED-4 and OLED-8). Thus, it may be presumed that F-Ph can transport electrons to the EML if electrons can be injected from the cathode to F-Ph.

To verify this hypothesis, the electron injection barriers (EIBs) at the Al/organic layer interfaces of each OLED were precisely investigated using a combination of LEIPS and ultraviolet photoelectron spectroscopy (UPS), as summarised from Fig. 1c–g (Supplementary Figs. 3 and 4, Supplementary Table 1). A clear difference in EIB was observed between the two OLEDs without an EIL: the EIB in OLED-5 is 1.56 eV, whereas that in OLED-1 is 0.22 eV, which is consistent with the relationship between the operating voltages of the two OLEDs (Supplementary Fig. 5). It is reasonable to assume that the smaller EIB in OLED-1 is caused by the coordination reaction between pyridine in F-Py and Al atoms that diffuse into the F-Py, as illustrated in Fig. 1h (Supplementary Fig. 6)^25,29^. The difference in WF between OLED-5 and OLED-1 (Δ_co_ in Fig. 1d) originates from the coordination reaction^25,26^. In addition, the direct measurement of EA by LEIPS clarified that the coordination reaction also causes the change in the EA of F-Py from 2.16 to 2.80 eV (δ_EA_ in Fig. 1d) (Supplementary Fig. 4)^30^. The EIB was found to be almost zero in OLED-2, where Liq was inserted between Al and F-Py, as shown in Fig. 1e. We see from the UPS/LEIPS spectra that no change in electronic structure was observed other than a change in WF with the addition of Li compounds into organic films, and WF was reduced by just depositing Li compounds onto ITO/ZnO (Supplementary Figs. 3 and 4). Thus, Li compounds such as Liq and LiF reduce the WF around the cathode simply because of their low charge neutrality level (Δ_Li_ in Fig. 1e and f)^31^. It can be concluded that the difference in operating voltage between OLED-2 and OLED-6 originates from the presence or absence of the coordination reaction. Note the EIB in OLED-8, the turn-on voltage of which is similar to that of OLED-2. The insertion of Py-hpp_2_ between Al and F-Ph reduces the WF to about 2.0 eV, resulting in an almost zero EIB. It was found that both the coordination reaction and the formation of H-bonds contribute to the production of this small WF, as illustrated in Fig. 1g and i (Supplementary Fig. 3). The deposition of 1-nm-thick Py-hpp_2_ on F-Ph decreases the surface WF to about 2.5 eV, which is caused by the formation of H-bonds (Supplementary Figs. 3, 7 and 8)^27^. The WF was further reduced to about 2.0 eV by utilising the coordination reaction when Al was deposited on this film (Supplementary Figs. 3 and 6, Supplementary Table 1)^25,26^. The importance of utilising both H-bonds and the coordination reaction can be seen from the characteristics of the OLED where hydrogen-free C_60_ was employed in the ETL (Supplementary Fig. 9). We see from Fig. 1b, c and g that the EIB difference of only 1.56 eV causes a turn-on voltage difference of about 10 V. Since the EA of ETL near the cathode is almost the same as the cathode WF in both OLED-8 and OLED-2, reducing the EIB to near zero seems to be essential to deliver electrons from the cathode to the EML at a low operating voltage. In addition, the results of our experiment suggest that nitrogen-containing heterocyclic compounds perform electron injection from the cathode rather than electron transport.

### Fabrication of over 100 OLEDs and their characteristics

**Fig. 2:**
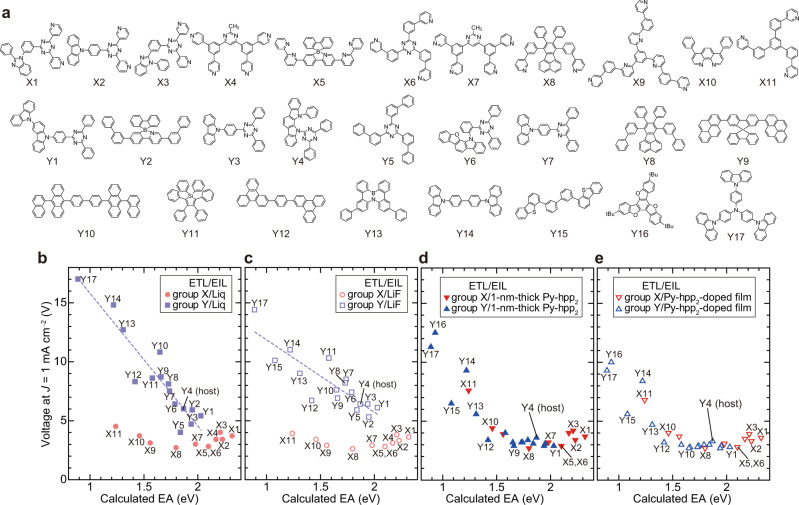
Summary of OLED characteristics using 28 materials as ETL. **a** Chemical structure of materials used as ETL in green phosphorescent OLEDs. **b**–**e** Summary of correlation between calculated EA of materials used as ETL (horizontal axis) and operating voltage of OLEDs at current density (*J*) of 1 mA cm^−2^ (vertical axis) (**b** EIL is Liq; **c** EIL is LiF; **d** EIL is 1-nm-thick Py-hpp_2_; **e** EIL is Py-hpp_2_-doped film). The luminance at *J* = 1 mA cm^−2^ is about 800 cd m^−2^ in almost all OLEDs.

To investigate the electron injection/transport mechanism, over 100 OLEDs were fabricated using the 28 materials shown in Fig. 2a for the ETL in Fig. 1a (Supplementary Figs. 10–17, Supplementary Tables 2 and 3). The materials shown in Fig. 2a are divided into two groups: one comprises typical ETMs with nitrogen-containing heterocycles (X1–X11, group X) and the other comprises materials not generally used for ETLs (Y1–Y17, group Y). In addition to the 1-nm-thick Liq, LiF, and Py-hpp_2_ used in Fig. 1, a 5-nm-thick Py-hpp_2_-doped film consisting of both Py-hpp_2_ and one of the materials shown in Fig. 2a was also used for the EIL. Since both the coordination reaction and the formation of H-bonds are effective only for reducing the EIB, the thick Py-hpp_2_-doped film sometimes increases the operating voltage of OLEDs, unlike typical n-doped systems (Supplementary Fig. 9)^8,9,12,25–27^. The operating voltages of OLEDs, which are highly dependent on the EIL/ETL combination, were analysed. Since the operating voltage is considered to be correlated with the energy-level alignment in the OLED from the results shown in Fig. 1, the operating voltage of each OLED at a current density of 1 mA cm^–2^ is plotted against the EA of each material estimated by DFT calculation (Fig. 2b, c, d and e). Although the EAs estimated from the DFT calculation are not accurate, they can be used as relative values for analysis (Supplementary Fig. 18). Note the difference in operating voltage between the OLEDs with group X and the OLEDs with group Y shown in Fig. 2b and c. The OLEDs with group X exhibited lower operating voltages, which originate from the coordination reaction between Al and nitrogen-containing heterocycles, as illustrated in Fig. 1. Although the operating voltages of the OLEDs with group Y shown in Fig. 2b and c are higher than those of the OLEDs with group X owing to a lack of the coordination reaction, it is natural that the least-squares approximations of the operating voltages are straight lines since Liq and LiF only reduce the WF around the cathode^31^. In contrast, the operating voltages of the OLEDs with group Y are significantly reduced by using Py-hpp_2_ as the EIL (Fig. 2d and e). The operating voltage is strongly correlated with the EA independently of the molecular structure, and a current density of 1 mA cm^–2^ was obtained at operating voltages below 5 V for the ETLs with a calculated EA above 1.3 eV (Y1–Y13), as shown in Fig. 2e. We see from Fig. 2d and e that it is not necessary to use typical ETMs to obtain OLED emission at a low operating voltage. Additionally, almost all OLEDs with Py-hpp_2_-doped films exhibit an external quantum efficiency (EQE) of about 20%, independent of the ETLs (Supplementary Figs. 16 and 17). It is concluded that the materials in group X, which were previously considered to be ETMs, are actually essential for electron injection rather than for electron transport.

**Fig. 3:**
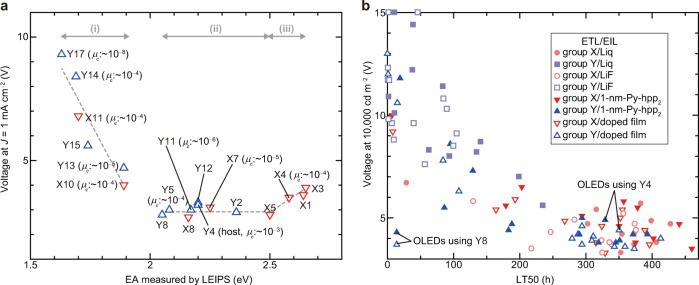
Quantitative analysis of OLED characteristics. **a** Summary of correlation between actual EA of materials used as ETL (horizontal axis) and operating voltage of OLEDs with Py-hpp_2_-doped film at *J* of 1 mA cm^−2^ (vertical axis). The electron mobility (*μ*_e_) of some materials is also shown. **b** Summary of correlation between operational lifetime (LT50) (horizontal axis) and operating voltage of OLEDs at luminance of 10,000 cd m^−2^ (vertical axis). LT50 is the time for the luminance to decay by 50% from 10,000 cd m^−2^.

To discuss the electron injection/transport mechanism in detail, the correlation between the operating voltages of OLEDs with Py-hpp_2_-doped films and the actual EA of several materials is summarised in Fig. 3a. For some of the materials, their reported electron mobilities are also described^13–17,32–35^. The operating voltage can be divided into three regions (i)–(iii). Since the WF is reduced to be about 2 eV by using Py-hpp_2_, it is likely that the linear increase in the operating voltage in region (i) is caused by the increase in EIB at the organic/cathode interface (Supplementary Fig. 19). In region (ii), where the EIB is almost zero, the operating voltage is low, independent of the molecular structure and electron mobility. Whereas in region (iii), the operating voltage increases, which can be attributed to the large difference in EA between the host (Y4) and the ETL^20^. Note the operating voltages of OLEDs with X4 and X7 with similar molecular structures. Although the electron mobility of X4 is one order of magnitude higher than that of X7, the OLED with X4 exhibits a higher operating voltage, which may originate from the large EA difference between X4 and Y4 (host)^13,20^. It was also confirmed that the operating voltages of the OLEDs with Y14 and Y17 are comparable even when the electron mobilities of Y14 and Y17 differ by four orders of magnitude^32,35^. Thus, the most stringent requirement to obtain OLED emission at a low operating voltage was found to be the minimisation of the energy barrier between the cathode and the EML.

Furthermore, the operational lifetime of OLEDs, which has been intensively studied in recent years, has been analysed with an unprecedented amount of lifetime data (Fig. 3b)^36–39^. With the exception of Y8, the lifetime is highly dependent on the operating voltage and almost independent of the material used in the EIL/ETL. Thus, it has been found that the main role of the EIL/ETL in long-lived OLEDs is to adjust the charge balance around the EML. Since the operating voltage and lifetime of many OLEDs with Py-hpp_2_ as part of the EIL are comparable to those of state-of-the-art green OLEDs reported in the literature, the range of choices of materials used between the cathode and the EML is expected to be increased greatly by our finding^18,19^. Among these OLEDs with low operating voltages and long lifetimes, the most noteworthy is a simplified OLED in which both the light-emitting host and the ETL are Y4. Thus, the application of Py-hpp_2_ to the EIL and the understanding of the electron injection/transport mechanism will cause a paradigm shift not only in the concept of electron injection/transport materials, but also in the structures of OLEDs. A significant advantage of Ph-hpp_2_ over 2,2′,2″-(1,3,5-benzinetriyl)-tris(1-phenyl-1-H-benzimidazole), which was reported to be a good EIL in a recent study, was also confirmed (Supplementary Fig. 20)^40^.

### Simply structured blue OLED

**Fig. 4:**
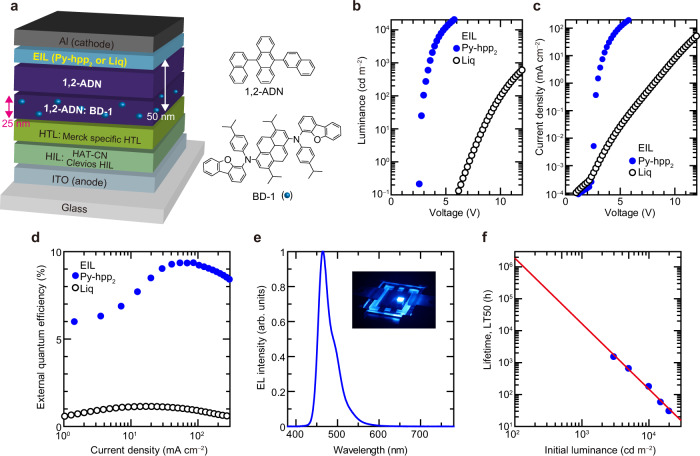
Demonstration of an innovative blue OLED. **a** Multilayer structure of the OLED, chemical structure of the materials used in the EML. **b**, **c** Luminance–voltage and current density–voltage characteristics of blue fluorescent OLEDs with different EILs. **d** EQE traces of devices with different EILs. **e** EL spectrum of a blue OLED with Py-hpp_2_. The inset is a photograph of the blue OLED. **f** Lifetime extrapolation for the blue OLED with Py-hpp_2_. Data points are obtained at different initial luminance values.

On the basis of these findings, we have succeeded in developing an innovative blue fluorescent OLED with low operating voltage, high efficiency and high operational stability despite its simple device structure (Fig. 4). Since the EAs of materials used in blue EMLs are generally smaller than those of materials used in green and red EMLs, it has been difficult to reduce the operating voltage of blue OLEDs, and improving their operational stability has also been the biggest challenge for their practical application. However, we see from Fig. 2d and e that Py-hpp_2_ can be used to efficiently inject electrons from the cathode into some aromatic hydrocarbons (Y9, Y10 and Y12) that are used in blue fluorescent OLEDs as the light-emitting host. Thus, we designed the simply structured OLED shown in Fig. 4a, where the emitting host and EIL are adjacent. Since the operating voltage was slightly lower for the blue OLED in which 1,4,5,8,9,11-hexaazatriphenylenehexacarbonitrile (HAT-CN) was deposited on top of ITO/Clevious HIL 1.3N (supplied by Heraeus Holding GmbH), we employed a stacked structure of Clevious HIL 1.3N and HAT-CN as the HIL of blue OLEDs (Supplementary Fig. 21). The emitting host 1,2-ADN is effective for improving the EQE by utilising TTA, and the fluorescent emitter BD-1 is a promising material for demonstrating long-lived OLEDs^2,41^. In recent years, doping of alkali elements has not been indispensable to realise red/green phosphorescent OLEDs with low operating voltages; however, alkali-element-doped ETL has been essential to reduce the operating voltage of blue fluorescent OLEDs owing to the small EA of blue EMLs^18,19,41^. The blue OLED consisting of a host and Py-hpp_2_ exhibits the lowest operating voltage among the blue OLEDs consisting of various EIL/ETL combinations including Liq-doped ETL (Supplementary Fig. 21)^41^. In particular, an EIL-dependent operating voltage was clearly observed in the simply structured OLED, as shown in Fig. 4a, b, and c: the luminance of the blue OLED was increased by about 200,000 times at an applied voltage of 6 V by changing the EIL from Liq to Py-hpp_2_. The blue OLED with Py-hpp_2_ exhibits a high EQE of more than 9%, realised by utilising TTA as shown in Fig. 4d^2,41^. In addition, the blue OLED with Py-hpp_2_ as the EIL shows both a good colour purity and an extremely long operational lifetime, as shown in Fig. 4e and f. Since the time for the luminance to decay to 50% of the initial luminance of 100 cd m^–2^, which is discussed as the lifetime for many blue OLEDs (LT50), is too long to be measured, we estimated the lifetime from the data shown in Fig. 4f (Supplementary Fig. 22)^36,42,43^. The lifetime was estimated to be more than 1,000,000 h, which is almost the same as that of OLEDs used in commercial products^36^. The time for the luminance to decay to 50% of the initial luminance of 1000 cd m^–2^, which is examined in recent blue OLEDs, is also expected to be long (about 6000 h) (Supplementary Fig. 23)^44^. The fact that a long-lived blue OLED could be realised without using typical ETLs is of great significance for the realisation of highly efficient and long-lived blue OLEDs, which is at the frontier of OLED research. Py-hpp_2_ will eliminate the need for a typical ETM with high triplet energy (E_T_) and stability, which is a bottleneck in realising long-lived blue OLEDs fabricated using phosphorescent and TADF emitters^45^. Considering the confinement of E_T_, one of the most promising materials as an alternative to the typical ETM is the emitting host, the E_T_ of which is basically higher than that of emitters, as demonstrated in Fig. 2. On the other hand, since high efficiencies have been obtained in this study and in previous studies even in green phosphorescent OLEDs with anthracene derivatives with low E_T_, we believe that a variety of materials can be used for ETLs, regardless of their E_T_, if electrons can be injected efficiently from good EILs such as Py-hpp_2_^19^.

## Discussion

We conclude that both the reactive alkali elements and typical ETMs, such as compounds with nitrogen-containing heterocycles, are not essential for delivering electrons from the cathode to the EML in OLEDs if electrons can be injected effectively. This liberation of materials used in OLEDs was achieved by tuning the WF near the cathode to about 2.0 eV using the superbase, and simply structured OLEDs that combine low operating voltage, high efficiency and high operational stability were realised. The fact that a WF equivalent to that of Cs, which has the smallest WF among the reactive alkali elements, is realised suggests that alkali elements near the cathode are unnecessary. The electron injection/transport mechanism unravelled in this study is expected to contribute to significant progress in organic electronics.

## Methods

### Fabrication of organic light-emitting diodes (OLEDs)

Green phosphorescent OLEDs (shown in Figs. 1, 2 and 3) were fabricated on glass substrates coated with a 100-nm-thick ITO layer. Prior to the fabrication of the organic layers, the substrate was cleaned using ultrapurified water, and organic solvents, and by UV–ozone treatment. After the UV–ozone treatment, Clevios HIL 1.3 N was spun onto the substrate to form a 10-nm-thick layer. Clevios HIL 1.3 N is effective for not only injecting holes from the ITO, but also reducing the possibility of electrical shorts within the device. The film structure of the green phosphorescent OLEDs was ITO (100 nm)/Clevios HIL 1.3 N (10 nm)/α-NPD (20 nm)/4DBFP3Q (10 nm)/DIC-TRZ:Ir(mppy)_3_ (3 wt%, 25 nm)/electron transport layer (ETL, 40 nm), where α-NPD is 4,4′-bis[N-(1-naphthyl)-N-phenyl-amino]biphenyl, 4DBFP3Q is N3,N3‴-bis(dibenzo[b,d]furan-4-yl)-N3,N3‴-diphenyl-[1,1′:2′,1″:2″,1‴-quaterphenyl]-3,3‴-diamine, DIC-TRZ is 2,4-diphenyl-6-bis(12-phenylindolo)[2,3-a] carbazol-11-yl)-1,3,5-triazine, and Ir(mppy)_3_ is *fac*-tris(3-methyl-2-phenylpyridinato-N,C2′-)iridium(III). In this study, we used various materials as the ETL, as shown in Figs. 1a and 2a. After the ETL was formed, an electron injection layer (EIL) was deposited. In the OLEDs, 1-nm-thick LiF, Liq, and Py-hpp_2_ were used as EILs. In addition to these three EILs, we employed a 5-nm-thick doped film consisting of 40 wt%-Py-hpp_2_ and another material used as the ETL. The evaporation rate of organic materials was about 0.04 nm/s, except for Py-hpp_2_ and emitter dopants (Supplementary Table 4). After the formation of the EIL, a 100-nm-thick Al layer was deposited as the cathode. The devices were encapsulated using a UV-epoxy resin and a glass cover in nitrogen atmosphere after cathode formation.

Blue fluorescent OLEDs (shown in Fig. 4) were fabricated similarly to the green phosphorescent OLEDs except for the thickness of the ITO layer (70 nm). The film structure of the blue fluorescent OLEDs was ITO (70 nm)/Clevios HIL 1.3 N (10 nm)/HAT-CN (5 nm)/Merck specific HTL (20 nm)/1,2-ADN:BD-1 (3 wt%, 25 nm)/ETL (25 nm). After the ETL was formed, the EIL was deposited. In the OLEDs shown in Fig. 4, 1-nm-thick Liq and Py-hpp_2_ were used as the EILs. After the formation of each EIL, a 100-nm-thick Al layer was deposited as the cathode. The devices were encapsulated using a UV-epoxy resin and a glass cover in nitrogen atmosphere after cathode formation.

Red phosphorescent OLEDs (Supplementary Fig. 9) were fabricated similarly to the green phosphorescent OLEDs except for the thickness of the ITO layer (150 nm). The film structure of the red phosphorescent OLEDs was ITO (150 nm)/Clevios HIL 1.3 N (10 nm)/α-NPD (40 nm)/Zn(BTZ)_2_:Ir(piq)_3_ (6 wt%, 30 nm)/ETL (45 nm), where Zn(BTZ)_2_ is bis[2-(2-hydroxyphenyl)benzothiazolato]zinc(II)] and Ir(piq)_3_ is tris[1-phenylisoquinolinato-C2,N]iridium(III). The materials used as ETLs were 6″-(4-([2,2′-bipyridin]-6-yl)-2-(5H-dibenzo[b,d]borolyl)phenyl)-2,2′:6′,3″-terpyridine (spB-BPy_2_, **X5** in Fig. 2a) and C_60_. After the ETL was formed, the EIL was deposited. In the OLEDs shown in Supplementary Fig. 9, 1-nm-thick LiF, Liq, and Py-hpp_2_ were used as the EILs. After the formation of each EIL, a 100-nm-thick Al layer was deposited as the cathode. The devices were encapsulated using a UV-epoxy resin and a glass cover in a nitrogen atmosphere after cathode formation.

### Device characterisation

Electroluminescence (EL) spectra and luminance were measured using a spectroradiometer (Minolta CS-1000). A digital SourceMeter (Keithley 2400) and a desktop computer were used to operate the devices. We assumed that the emission from the OLEDs was isotropic, such that the luminance was Lambertian; thus, we calculated the external quantum efficiency (EQE) from the luminance, current density, and EL spectra.

### Ultraviolet photoelectron spectroscopy (UPS)

Glass substrates coated with a 150-nm-thick ITO layer were cleaned with ultrapurified water and organic solvents, and by UV–ozone treatment. ZnO was deposited using a Mirror Tron sputtering system (Choshu Industry Co., Ltd.). Then, we deposited organic thin films and/or metals using a vacuum evaporation system, and the samples were exposed to air immediately. The samples were placed in a holder and then introduced into the load lock chamber of a UPS measurement apparatus. The total time of exposure to air was 10 min for all the samples. UPS spectra of the samples were measured using a CHA analyser with a 128-channel detector; the excitation source was a HeI (21.22 eV) discharge lamp. A bias of −8.0 V was applied to each sample to separate the sample and the secondary edge for the analyser.

### Low-energy inverse photoemission spectroscopy (LEIPS)

LEIPS spectra of 5-nm-thick host films on glass/ITO were measured using an LEIPS measurement system (ALS Technology Co., Ltd.). No discernible dependence on the sample current or photon energy was observed, confirming that the LEIPS spectra were free from sample charging. The electron affinity was obtained by the standard procedure for determining the ionisation energy by UPS. The onset of the LEIPS spectrum was determined as the intersection of the straight line fitted to the onset region of the spectrum and the baseline (Supplementary Fig. 4).

### DFT calculation

Quantum chemical calculations were performed using the hybrid density functional theory (DFT) functional, Becke and Hartree–Fock exchange, and the Lee, Yang and Parr correlation (B3LYP) as implemented by the Gaussian 09 program packages. Electrons were described by Pople’s 6-31 G(d,p) basis sets for molecular structure optimisation.

## Supplementary information

Supplementary Information

## Data Availability

The data that support the plots within the paper are available from the corresponding author upon reasonable request.

## References

[1] Tang CW, VanSlyke SA (1987). Organic electroluminescent diodes. Appl. Phys. Lett..

[2] Kondakov DY, Pawlik TD, Hatwar TK, Spindler JP (2009). Triplet annihilation exceeding spin statistical limit in highly efficient fluorescent organic light-emitting diodes. J. Appl. Phys..

[3] Baldo MA (1998). Highly efficient phosphorescent emission from organic electroluminescent devices. Nature.

[4] Uoyama H, Goushi K, Shizu K, Nomura H, Adachi C (2012). Highly efficient organic light-emitting diodes from delayed fluorescence. Nature.

[5] Noda H (2019). Critical role of intermediate electronic states for spin-flip processes in charge-transfer-type organic molecules with multiple donors and acceptors. Nat. Mater..

[6] Tang, X. et al. Highly efficient luminescence from space-confined charge-transfer emitters. *Nat. Mater*. **19**, 1332–1338 (2020). 10.1038/s41563-020-0710-z 32541938

[7] Hung LS, Tang CW, Mason MG (1997). Enhanced electron injection in organic electroluminescence devices using an Al/LiF electrode. Appl. Phys. Lett..

[8] Walzer K, Maennig B, Pfeiffer M, Leo K (2007). Highly efficient organic devices based on electrically doped transport layers. Chem. Rev..

[9] Lin X (2017). Beating the thermodynamic limit with photo-activation of n-doping in organic semiconductors. Nat. Mater..

[10] Bin Z, Liu Z, Qiu Y, Duan L (2018). Efficient n-dopants and their roles in organic electronics. Adv. Opt. Mater..

[11] Xiao L (2011). Recent progresses on materials for electrophosphorescent organic light-emitting devices. Adv. Mater..

[12] Lüssem B, Riede M, Leo K (2013). Doping of organic semiconductors. Phys. Stat. Sol. a.

[13] Sasabe H, Kido J (2011). Multifunctional materials in high-performance OLEDs: challenges for solid-state lighting. Chem. Mater..

[14] Chen H-F (2009). 1,3,5-Triazine derivatives as new electron transport–type host materials for highly efficient green phosphorescent OLEDs. J. Mater. Chem..

[15] Su S-J, Takahashi Y, Chiba T, Takeda T, Kido J (2009). Structure-property relationship of pyridine-containing triphenyl benzene electron-transport materials for highly efficient blue phosphorescent OLEDs. Adv. Funct. Mater..

[16] Matsushima T (2010). High electron mobility layers of triazines for improving driving voltages, power conversion efficiencies, and operational stability of organic light-emitting diodes. Org. Electron..

[17] Yu G (2005). Structures, electronic states, photoluminescence, and carrier transport properties of 1,1-disubstituted 2,3,4,5-tetraphenylsiloles. J. Am. Chem. Soc..

[18] Bian M (2018). Positional isomerism effect of spirobifluorene and terpyridine moieties of “(A)n–D–(A)n” type electron transport materials for long-lived and highly efficient TADF-PhOLEDs. J. Mater. Chem. C..

[19] Zhang D, Qiao J, Zhang D, Duan L (2017). Ultrahigh-efficiency green PHOLEDs with a voltage under 3 V and a power efficiency of nearly 110 lm W^–1^ at luminance of 10 000 cd m^–2^. Adv. Mater..

[20] Yadav RAK, Dubey DK, Chen S-Z, Liang T-W, Jou J-H (2020). Role of molecular orbital energy levels in OLED performance. Sci. Rep..

[21] Yoshida H (2015). Principle and application of low energy inverse photoemission spectroscopy: a new method for measuring unoccupied states of organic semiconductors. J. Electron Spectrosc. Relat. Phenom..

[22] Yoshida H, Yoshizaki K (2015). Electron affinities of organic materials used for organic light-emitting diodes: a low-energy inverse photoemission study. Org. Electron..

[23] Zhou Y (2012). A universal method to produce low-work function electrodes for organic electronics. Science.

[24] Tang CG (2019). Multivalent anions as universal latent electron donors. Nature.

[25] Bin Z (2019). Making silver a stronger n-dopant than cesium via in situ coordination reaction for organic electronics. Nat. Commun..

[26] Fukagawa H (2020). Understanding coordination reaction for producing stable electrode with various low work functions. Nat. Commun..

[27] Fukagawa H (2019). Universal strategy for efficient electron injection into organic semiconductors utilising hydrogen bonds. Adv. Mater..

[28] Schwamm RJ (2016). ^15^N NMR spectroscopy, X-ray and neutron diffraction, quantum-chemical calculations, and UV/vis-spectrophotometric titrations as complementary techniques for the analysis of pyridine-supported bicyclic guanidine superbases. J. Org. Chem..

[29] Lee JH, Yi Y, Moon DW (2008). Direct evidence of Al diffusion into tris-(8-hydroquinoline) aluminum layer: medium energy ion scattering analysis. Appl. Phys. Lett..

[30] Isshiki Y, Fujii S, Nishino T, Kiguchi M (2018). Fluctuation in interface and electronic structure of single-molecule junctions investigated by current versus bias voltage characteristics. J. Am. Chem. Soc..

[31] Park S, Yi Y, Cho SW, Lee H (2016). Work function reduction using 8-hydroxyquinolinolato-lithium for efficient inverted devices. Chem. Phys. Lett..

[32] Xiang C, Koo W, So F, Sasabe H, Kido J (2013). A systematic study on efficiency enhancements in phosphorescent green, red and blue microcavity organic light emitting devices. Light. Sci. Appl.

[33] Hashimoto S (2014). Triplet-energy control of polycyclic aromatic hydrocarbons by BN replacement: development of ambipolar host materials for phosphorescent organic light-emitting diodes. Chem. Mater..

[34] Zhang D (2014). High-efficiency fluorescent organic light-emitting devices using sensitizing hosts with a small singlet-triplet exchange energy. Adv. Mater..

[35] Kubo S, Kaji H (2018). Parameter-free multiscale simulation realising quantitative prediction of hole and electron mobilities in organic amorphous system with multiple frontier orbitals. Sci. Rep..

[36] Zhang Y, Lee J, Forrest SR (2014). Tenfold increase in the lifetime of blue phosphorescent organic light-emitting diodes. Nat. Commun..

[37] Lee J (2017). Hot excited state management for long-lived blue phosphorescent organic light-emitting diodes. Nat. Commun..

[38] Cui LS (2017). Long-lived efficient delayed fluorescence organic light-emitting diodes using n-type hosts. Nat. Commun..

[39] Kim S (2018). Degradation of blue-phosphorescent organic light-emitting devices involves exciton-induced generation of polaron pair within emitting layers. Nat. Commun..

[40] Kotadiya NB, Blom PWM, Wetzelaer G-JAH (2019). Efficient and stable single-layer organic light-emitting diodes based on thermally activated delayed fluorescence. Nat. Photon.

[41] Sato T, Miyamae T, Ohata H, Tsutsui T (2019). Direct observations of the charge behavior of a high-efficiency blue organic light-emitting diode under operating conditions using electric-field-induced doubly resonant sum-frequency-generation vibrational spectroscopy. Org. Electron..

[42] Féry C, Racine B, Vaufrey D, Doyeux H, Cinà S (2005). Physical mechanism responsible for the stretched exponential decay behavior of aging organic light-emitting diodes. Appl. Phys. Lett..

[43] Orselli E, Maunoury J, Bascour D, Catinat J-P (2012). Orange phosphorescent organic light-emitting diodes with high operational stability. Org. Electron..

[44] Chan CY (2021). Stable pure-blue hyperfluorescence organic light-emitting diodes with high-efficiency and narrow emission. Nat. Photon.

[45] Kondo Y (2019). Narrowband deep-blue organic light-emitting diode featuring an organoboron-based emitter. Nat. Photon.

