# Supplementary material for: Unravelling the electron injection/transport mechanism in organic light-emitting diodes
Source: Nat Commun. 2021 May 11;12:2706. doi: 10.1038/s41467-021-23067-2 (PMC8113438; doi:10.1038/s41467-021-23067-2)
Supplement: Supplementary file 1 — Supplementary Information [file 41467_2021_23067_MOESM1_ESM.pdf]

SUPPLEMENTARY INFORMATION

Unravelling the electron injection/transport mechanism in organic light-emitting diodes

Tsubasa Sasaki[1], Munehiro Hasegawa[2], Kaito Inagaki[3], Hirokazu Ito[3], Kazuma Suzuki[3], Taku Oono[1], Katsuyuki Morii[2,4], Takahisa Shimizu[1] and Hirohiko Fukagawa[1,*]

[1] Japan Broadcasting Corporation (NHK), Science & Technology Research Laboratories, 1-10-11 Kinuta, Setagaya-ku, Tokyo 157-8510, Japan
[2] Nippon Shokubai Co., Ltd., 5-8 Nishi Otabi-cho, Suita, Osaka 564-8512, Japan
[3] Tokyo University of Science, 1–3 Kagurazaka, Tokyo 162-8610, Japan
[4] Nippon Shokubai Research Alliance Laboratories, Osaka University, Osaka 565-0871, Japan

*e-mail. Email: fukagawa.h-fe@nhk.or.jp

Supplementary Notes

Materials:

OLED materials were mainly purchased from Luminescence Technology Corporation (Taiwan) and used after sublimation. X4 and X7 shown in Fig. 2a were purchased from FLASK Corporation. Y11 and Y15 shown in Fig. 2a were purchased from Sigma-Aldrich. Although many of the materials shown in Fig. 2 were previously reported [X2[1], X5[2], X6[3], X7[3], X11[3], Y1[4], Y3[2], Y4[5], Y5[3], Y7[1], Y13[6], Y15[7]], several novel compounds have been newly synthesised.

A superbase named 2,6-Bis(1,3,4,6,7,8-tetrahydro-2H-pyrimido[1,2-a]pyrimidin-1-yl)pyridine (Py-hpp$_2$) was synthesised according to the following procedure.

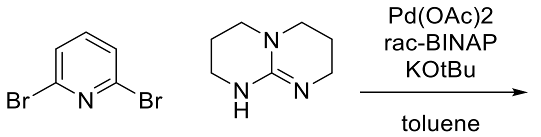

rac-BINAP (1.87 g, 3.0 mmol) and toluene (160 mL) were charged in an oven-dried round-bottomed flask and heated at 90 °C to dissolve the BINAP. The mixture was cooled to room temperature and $Pd(OAc)_2$ (450 mg, 2.0 mmol) was added and stirred for 30 min. To the resulting solution, 2,6-dibromopyridine (11.9 g, 50 mmol), 1,3,4,6,7,8-hexahydro-2H-pyrimido[1,2-a]pyrimidine (15.3 g, 110 mmol), and *t*-BuOK (15.7g, 140 mmol) were added and the reaction mixture was stirred at 90 °C overnight. After cooling to room temperature, diethyl ether was added to the reaction mixture and the mixture was filtered through Celite. After the evaporation of the filtrate, the residue was purified by recrystallisation from dibutyl ether to obtain Py-hpp2 as a pale yellow solid (13.0 g, 36.8 mmol, 74%).

^{1}H NMR (600 MHz, $CDCl_3$) δ 1.87 (quin, *J* = 5.87 Hz, 3H), 1.96 (quin, *J* = 6.24 Hz, 4H), 3.15 (t, *J* = 6.46 Hz, 4H), 3.21 (t, *J* = 6.02 Hz, 3H), 3.41 (t, *J* = 5.58 Hz, 4H), 3.84 (t, *J* = 6.0 Hz, 4H), 7.12 (d, *J* = 8.22 Hz, 2H), 7.34 (t, *J* = 8.08 Hz, 1H).

1-(4-(4,6-Tris(2-pyridy)-1,3,5-triazin-2-yl)-9-phenyl-1H-benzo[d]imidazole (X1 shown in Fig. 2a) was synthesised according to the following procedure.

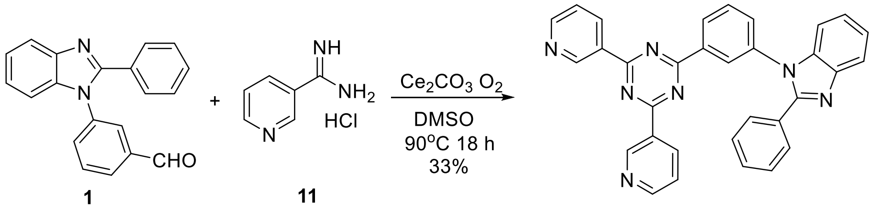

1 **11**

1 (1.26 g, 4.22 mol, 0.5 eq.), **11** (1.33 g, 8.45 mmol, 1.0 eq.), caesium carbonate (5.74 g, 16.9 mol, 2.0 eq.), and DMSO (13 mL) were added to a 1 L reactor. In an oxygen atmosphere, the suspension temperature was raised to a bath temperature of 90°C and the suspension was stirred at the same temperature for 24 h. To this suspension, tap water (100 mL) and ethyl acetate (50 mL) were added and stirred sufficiently, and then the suspension was divided into two layers. The aqueous layer was extracted with ethyl acetate (50 mL) and the organic layer was washed sequentially with tap water and saturated saline. The organic layer was concentrated to obtain a beige solid (1.84 g). After column purification (NH-SiO_2=50 g, chloroform/heptane = 1/1 -> chloroform only), the beige solid was purified again (SiO_2=50 g, hexane/ethyl acetate = 1/2 -> ethyl acetate only) to obtain a pale beige powder (1.15 g). This was heated, dispersed and washed with ethanol to yield a colourless powder (0.76 g, HPLC 96%). It was purified by preparative GPC and was again washed with ethanol by heating, dispersing and washing, and dried under high vacuum to give X1 (0.72 g, 1.43 mmol, 33%), a colourless solid.

^{1}H-NMR ($CDCl_3$) δ: 9.88 (s, 2H), 8.96–8.84 (m, 5H), 8.75 (t, J = 1.8 Hz, 1H), 7.96 (d, J = 7.7 Hz, 1H), 7.74 (t, J = 7.9 Hz, 1H), 7.69–7.62 (m, 2H), 7.57–7.47 (m, 3H), 7.45–7.28 (m, 6H).

9-(4-(4,6-Tris(2-pyridy)-1,3,5-triazin-2-yl)-1-phenyl-1H-benzo[d]imidazole (X3 shown in Fig. 2a) was synthesised according to the following procedure.

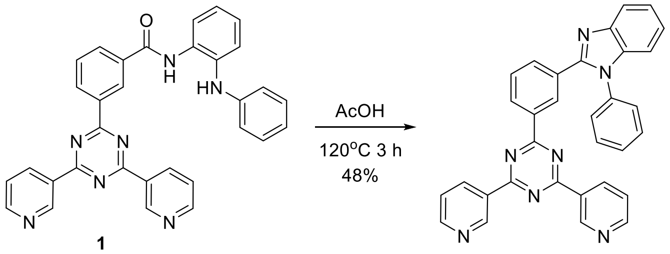

1 (1.31 g, 2.51 mmol, 1.0 eq.) and acetic acid (13 mL) were added to a 100 mL reactor. This solution was stirred at 120 °C for 3 h, released and cooled, and then added to an aqueous sodium 2N-hydroxide solution (200 mL) and chloroform (100 mL). This suspension was stirred at about 40 °C for 1 hour then filtered. The filtrate was divided into two layers, namely, the water layer, which was extracted with chloroform (50 mL), and the organic layer, which was washed with tap water and saturated saline, dried with anhydrous sodium sulfate, and concentrated to obtain a red mucilaginous liquid (1.21 g). This liquid was column-purified (NH-SiO_2: 50 g, chloroform/heptane = 2/1 -> 1/1) to obtain a red solid (1.1 g). This solid was purified by preparative GPC to obtain a light

yellow solid (0.72 g). X3 (0.61 g, 1.21 mmol, 48%), a colourless powder, was obtained by dispersing and washing the solid with ethanol and drying it under high vacuum.
^{1}H-NMR (400 MHz, $CDCl_3$): δ 9.81 (dd, 2H, J = 2.2, 0.7 Hz), 8.90 (dt, 2H, J = 8.0, 2.0 Hz), 8.87 (dd, 2H, J = 4.9, 1.7 Hz), 8.81 (t 1H, J = 1.6 Hz), 8.76 (dt, 1H, J = 7.9, 1.5 Hz), 8.09 (dt, 1H, J = 7.7, 1.5 Hz), 7.95 (d, 1H, J = 8.0 Hz), 7.63–7.57 (m, 3H), 7.55–7.44 (m, 5H), 7.42–7.36 (1H, m), 7.34–7.31 (m, 2H).

7,10-bis(3-(pyridin-3-yl)phenyl)-8,9-diphenylfluoranthene (X8 shown in Fig. 2a) was synthesised according to the following procedure.

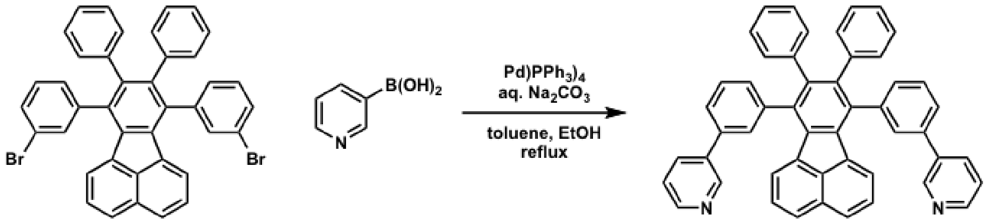

A mixture of 7,10-bis(3-bromophenyl)-8,9-diphenylfluoranthene (1.20 g, 1.80 mmol), 3-pyridineboronic acid (0.74 g, 6.9 mmol), $Pd(PPh_3)_4$ (0.12 g, 0.18 mmol), 2M aq. sodium carbonate (7.2 mL), toluene (30 mL), and ethanol (7.2 mL) was stirred under reflux overnight in nitrogen atmosphere. After cooling to room temperature, the reaction mixture was poured into water and then extracted with ethyl acetate. The combined organic extracts were washed with brine, dried over $MgSO_4$, and evaporated. The residue was purified by silica gel chromatography to obtain 7,10-bis(3-(pyridin-3-yl)phenyl)-8,9-diphenylfluoranthene (X8) as a white powder (0.77 mg, 1.17 mmol, 65%).
^{1}H NMR (600 MHz, $CDCl_3$) δ (ppm) 6.82 (dd, *J*=8.95, 7.19 Hz, 1H) 6.87–7.03 (m, 5H) 7.26–7.37 (m, 2H) 7.42–7.58 (m, 4H) 7.68–7.82 (m, 2H) 8.50–8.62 (m, 1H) 8.66–8.76 (m, 1H).

5-(biphenyl-3-yl)-2-(4-(biphenyl-3-yl)-2-(5H-dibenzo[b,d]borolyl)phenyl)pyridine (Y2 shown in Fig. 2a) was synthesised according to the following procedure.

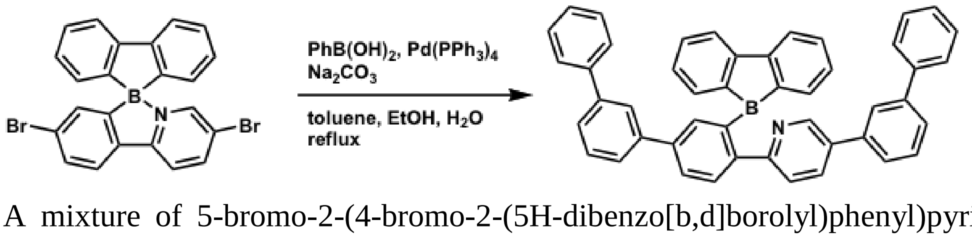

A mixture of 5-bromo-2-(4-bromo-2-(5H-dibenzo[b,d]borolyl)phenyl)pyridine (1.5 g, 3.17 mmol), 3-biphenylboronic acid (1.88 g, 9.51 mmol), $Pd(PPh_3)_4$ (0.11 g, 0.951 mmol),

sodium carbonate (1.68 g, 15.9 mmol), toluene (15 mL), ethanol (10 mL), and water (15 mL) was stirred under reflux overnight in argon atmosphere. After cooling to room temperature, toluene and water were added. The organic phase was separated and then the aqueous phase was extracted with toluene. The combined organic extracts were washed with brine, dried over Na_2SO_4, and evaporated. The residue was purified by silica gel chromatography to obtain 5-(biphenyl-3-yl)-2-(4-(biphenyl-3-yl)-2-(5H-dibenzo[b,d]borolyl)phenyl)pyridine (Y2) as a pale yellow solid (1.61 g, 2.59 mmol, 81%).

^{1}H NMR (600 MHz, C_6D_6) δ (ppm) 6.51–6.60 (m, 1H) 6.79 (t, *J*=7.78 Hz, 1H) 7.09–7.43 (m, 23H) 7.69 (dd, *J*=7.92, 1.76 Hz, 1H) 7.77 (t, *J*=1.76 Hz, 1H) 7.80 (d, *J*=8.22 Hz, 1H) 7.92–8.01 (m, 3H) 8.23 (t, *J*=1.17 Hz, 1H).

12-(2-4,6-diphenyl-1,3,5-triazin-2-yl)phenyl)-12H-benzofuro[2,3-a]carbazole (Y6 shown in Fig. 2a) was synthesised according to the following procedure.

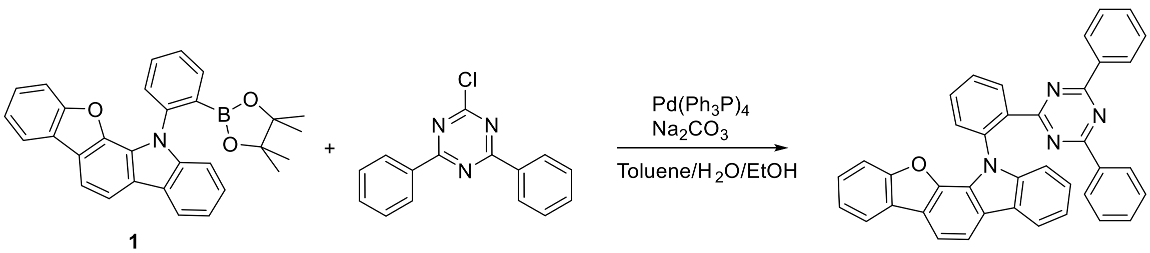

To the 100 mL reactor, **1** (0.8 g, 1.74 mmol, 1.0 eq.), 2-chloro-4,6-diphenyl-1,3,5-triazine (0.932 g, 3.48 mmol, 2.0 eq.), $Pd(Ph_3P)_4$ (0.1 g, 0.087 mmol, 0.05 eq.), $Pd(dppf)Cl_2$ (64 mg, 0.087 mmol, 0.05 eq.), sodium carbonate (0.554 g, 5.22 mmol, 3.0 eq.), toluene (16 mL), distilled water (16 mL), and ethanol (8 mL) were added. This suspension heated in a bath to a temperature of 90 °C and stirred at the same temperature for 18 hours. The suspension was filtered through cellulite, 5% ammonium chloride solution (15 mL) was added to the filtrate, then the filtrate was extracted with toluene (20 mL), washed with tap water and saturated saline, dried with anhydrous sodium sulfate, and concentrated to obtain a brown mucilaginous liquid (3.5 g). This liquid was column-purified (SiO_2=200 g, hexane/toluene=2/1 -> 1/1) to obtain a light green solid (1.29 g). The obtained solid and concentrated filtrate were purified by preparative GPC, and the resulting solids were heat-dispersed, washed with toluene and ethanol, and dried under a high vacuum to obtain the yellowish-green powder Y6 (0.96 g, 1.70 mmol, 78%).

^{1}H-NMR (400 MHz, $CDCl_3$): δ 8.67–8.64 (m, 1H, J = 7.5, 1.2 Hz), 8.07 (d, 1H, J = 7.8 Hz), 8.01 (d, 1H, J = 8.0 Hz), 7.94–7.80 (m, 8H), 7.77 (d, 1H, J = 8.0 Hz), 7.44–7.37 (m, 3H), 7.35–7.20 (m, 9H).

7,10-bis(biphenyl-3-yl)-8,9-diphenylfluoranthene (Y8 shown in Fig. 2a) was synthesised according to the following procedure.

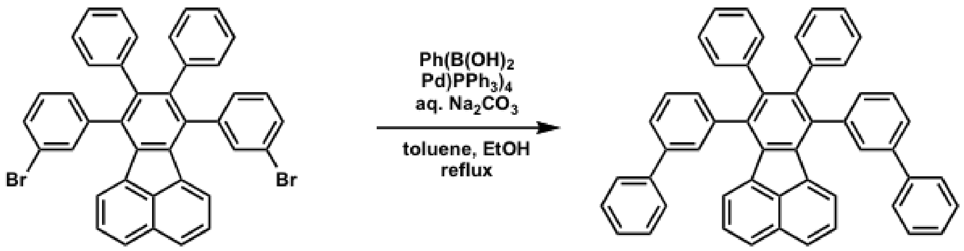

A mixture of 7,10-bis(3-bromophenyl)-8,9-diphenylfluoranthene (1.0 g, 1.51 mmol), 3-pyridineboronic acid (0.55 g, 5.52 mmol), $Pd(PPh_3)_4$ (0.087 g, 0.075 mmol), 2M aq. sodium carbonate (5.4 mL), toluene (15 mL), and ethanol (5.4 mL) was stirred under reflux overnight in nitrogen atmosphere. After cooling to room temperature, the reaction mixture was poured into water and then extracted with chloroform. The combined organic extract was washed with brine, dried over $MgSO_4$, and evaporated. The residue was purified by silica gel chromatography to obtain 7,10-bis(biphenyl-3-yl)-8,9-diphenylfluoranthene (Y8) as a white powder (696 mg, 1.06 mmol, 70%).

^{1}H NMR (600 MHz, $CDCl_3$) δ (ppm) 6.80 (d, *J*=7.04 Hz, 1H), 6.86–7.03 (m, 5H), 7.30–7.36 (m, 3H), 7.37–7.44 (m, 3H), 7.48–7.56 (m, 3H), 7.62 (dt, *J*=3.30, 1.72 Hz, 1H), 7.74 (d, *J*=7.92 Hz, 1H).

Supplementary Figures

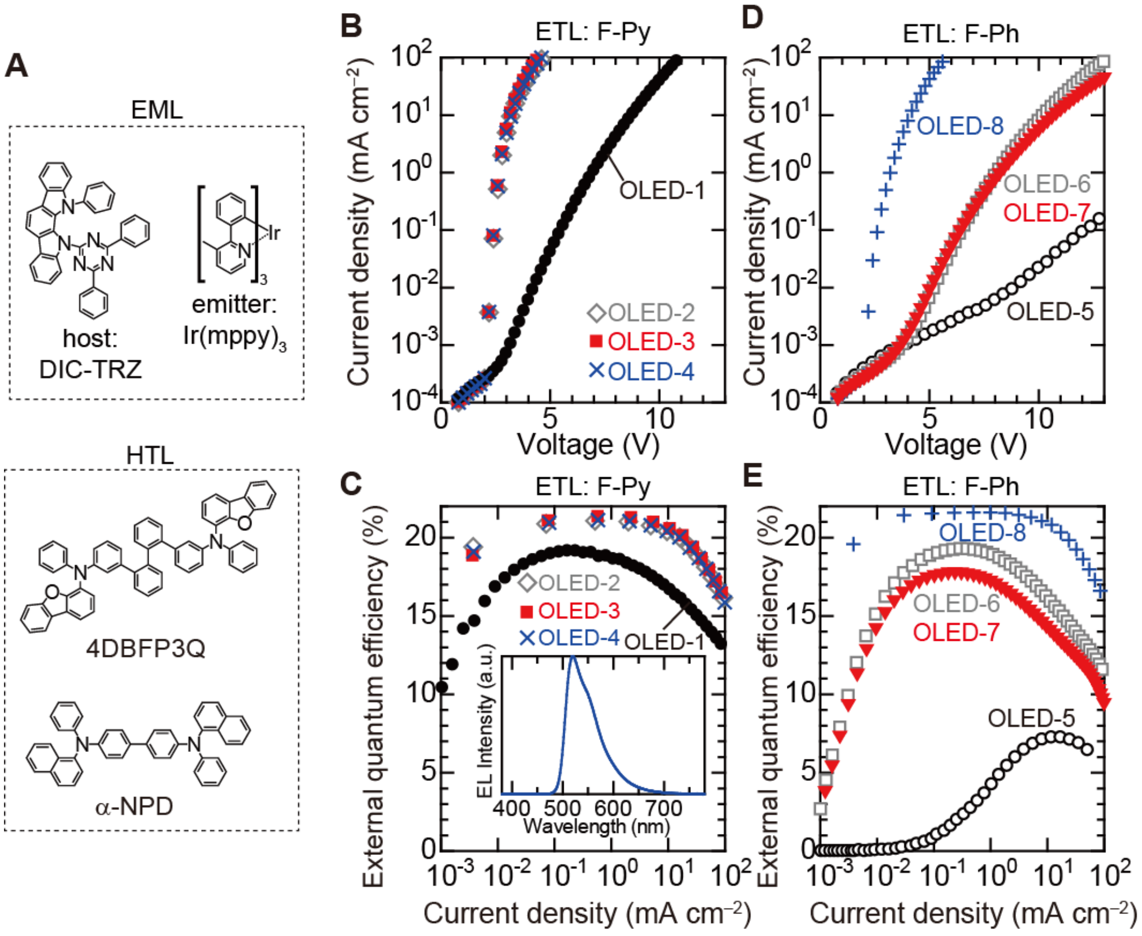

Supplementary Figure 1 | (A) Chemical structure of the materials used in the OLEDs. (B, D) Current density–voltage characteristics of OLEDs fabricated using F-Py and F-Ph as ETLs. (C) EQE–current density curves of OLEDs fabricated using F-Py as ETL. Inset: EL spectrum of OLED with Py-hpp$_2$ as EIL. (E) EQE–current density curves of OLEDs fabricated using F-Ph as ETL.

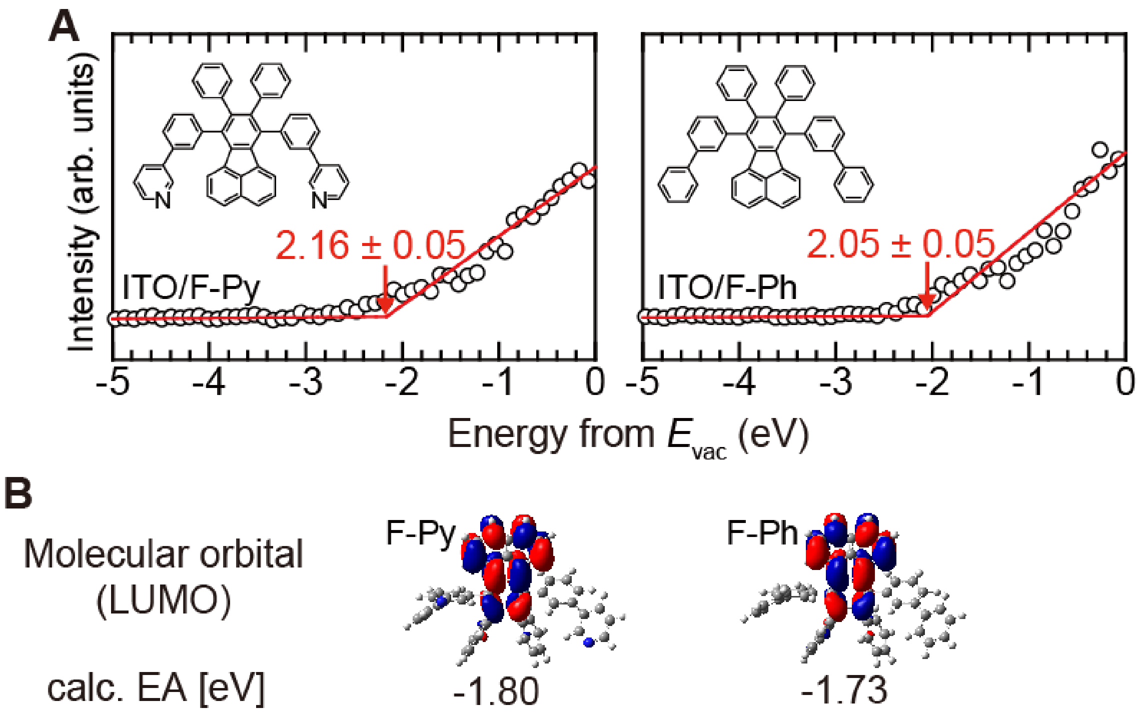

Supplementary Figure 2 | (A) LEIPS spectra taken at 260 nm for the films on ITO. The thickness of F-Ph and F-Py is 5 nm. The electron affinities indicated by arrows were estimated using the "onset of slope" application in ORIGN® 2020 software. (B) DFT calculation results for F-Ph and F-Py such as the lowest unoccupied molecular orbital (LUMO) and electron affinity (EA).

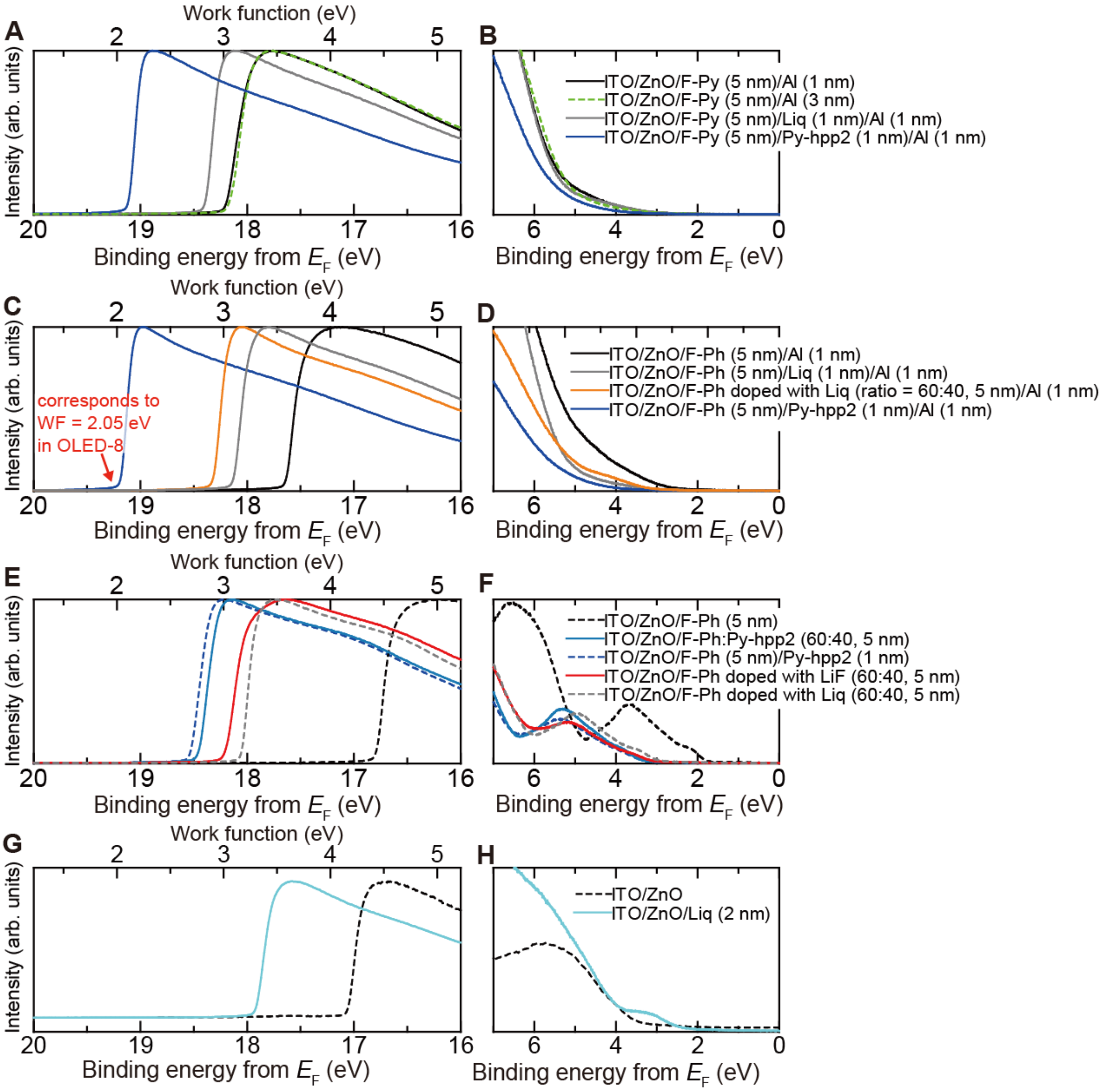

Supplementary Figure 3 | HeI UPS spectra of each film in the (A, C, E, G) secondary and (B, D, F, H) highest occupied molecular orbital (HOMO) regions. The actual work function (WF) around the top Al cathode/(EIL)/ETL (F-Ph or F-Py) can be seen from the cutoff positions of the UPS spectra shown in A and C. The effects of various EILs on the WF and valence band can be seen from the UPS spectra shown in E and F. WFs and ionisation potentials (IPs) estimated from the UPS spectra are summarised in Table S1. We see from A and B that the effect of the Al thickness (1 or 3 nm) on the electronic structure around the cathode is small. Although the WFs become smaller with the inclusion of LiF, Liq, and Py-hpp$_2$ (E), not only does the spectral shape of the HOMO not change, but also no gap state appears in all spectra (F). Additionally, the reduction of WF was reduced by just depositing Liq onto ITO/ZnO (G). Thus, it is reasonable to assume that Li compounds such as Liq and LiF reduce the WF around the cathode simply because of their low charge neutrality level[8].

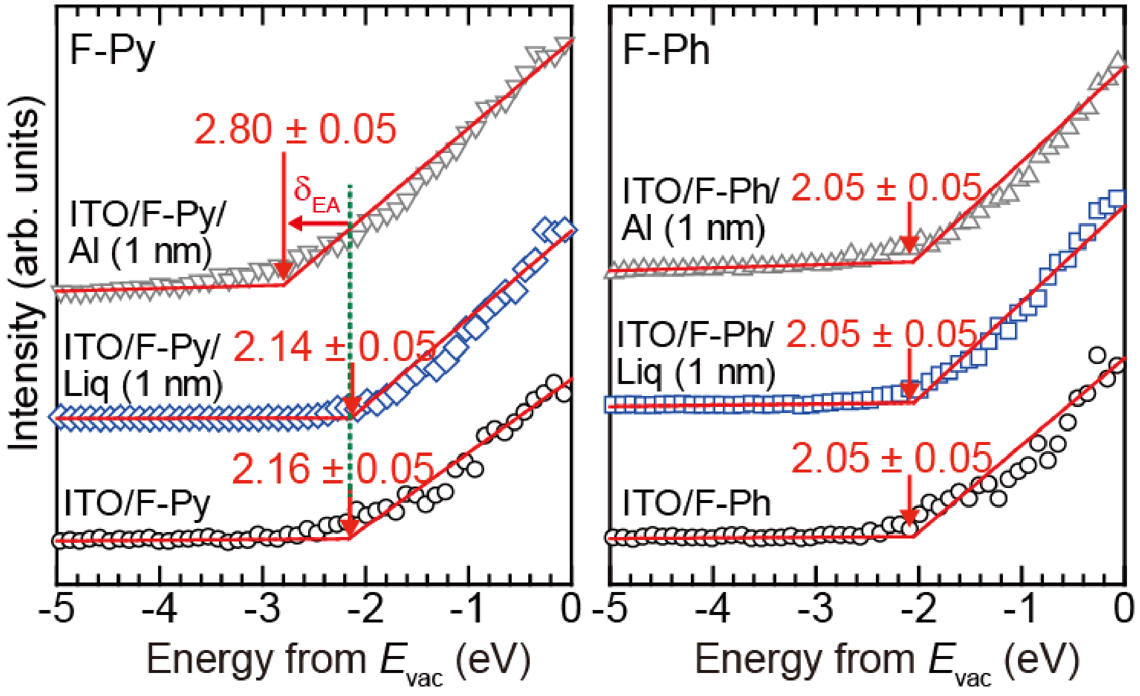

Supplementary Figure 4 | LEIPS spectra taken at 260 nm for the films on ITO. The thickness of F-Ph and F-Py is 5 nm, and 1-nm-thick Liq or Al is deposited on it. The electron affinities indicated by arrows were estimated using the "onset of slope" application in ORIGN® 2020 software. The results of our experiment clearly demonstrate that only the coordination reaction between the pyridine substituent and Al causes the change in EA (δ_{EA})[9]. It was also found that the effect of Li compounds on the EA of another organic compound is small.

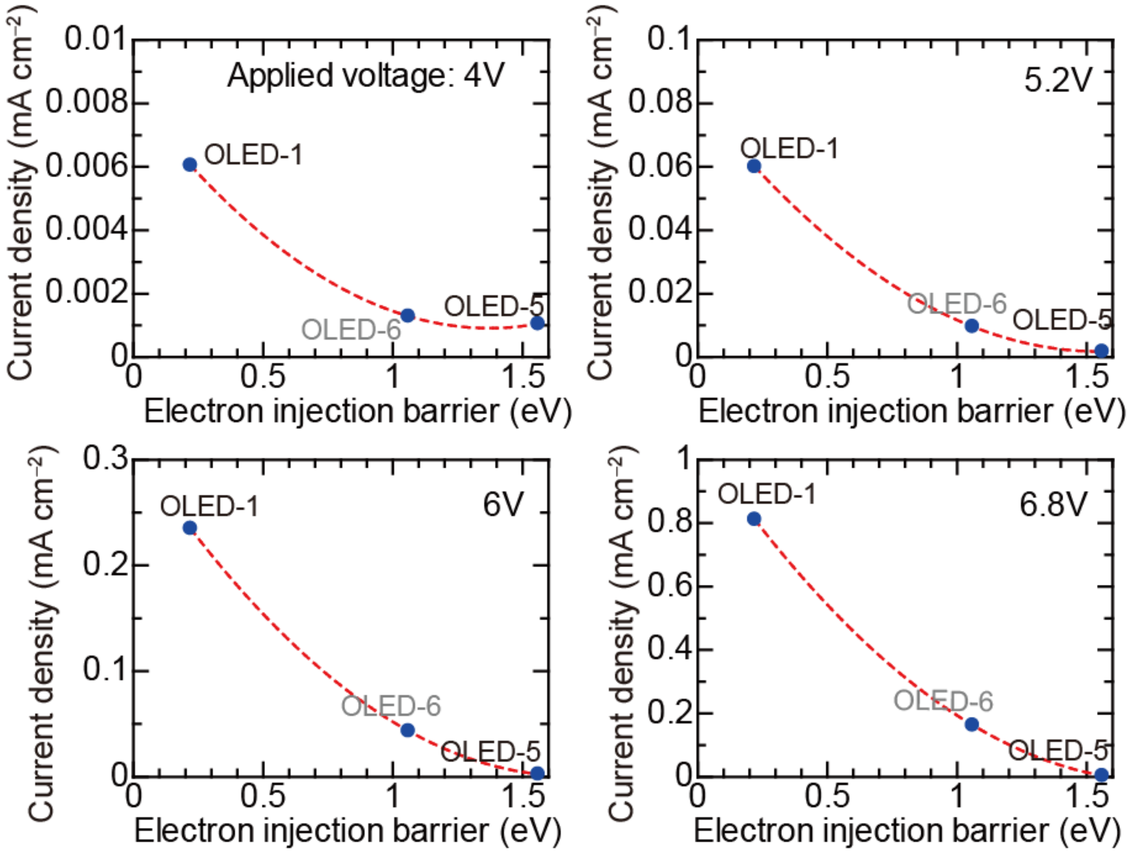

Supplementary Figure 5 | Correlation between the current density (vertical axis) and the electron injection barrier around each cathode (horizontal axis) for the three OLEDs with relatively high operating voltages shown in Fig. 1b. The reason for showing the current density instead of the luminance on the vertical axis is that the efficiencies of the three OLEDs are different as shown in Supplementary Figure 1.

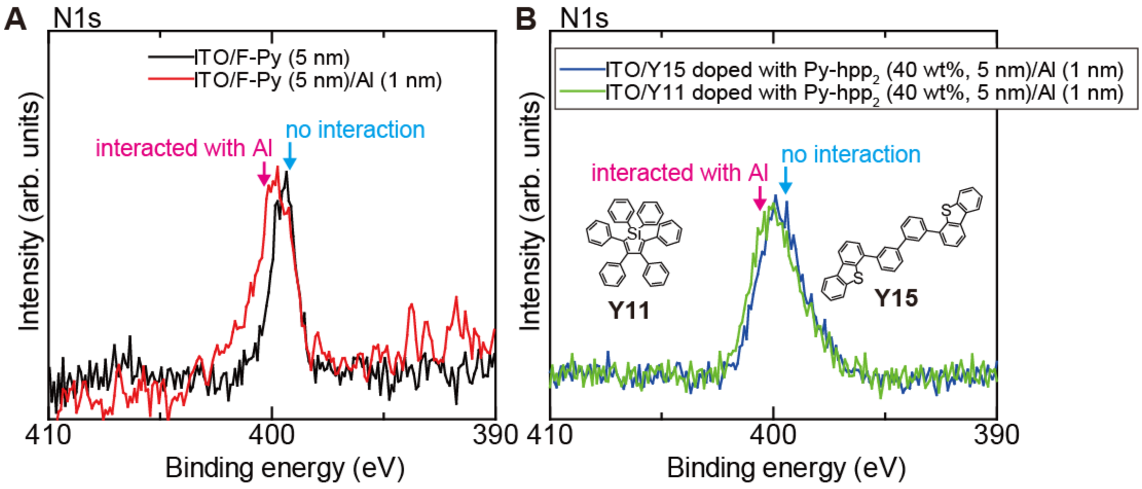

Supplementary Figure 6 | X-ray photoelectron spectroscopy spectra of (A) ITO/F-Py and ITO/F-Py/Al films, (B) ITO/Py-hpp$_2$ with different host/Al films[10,11].

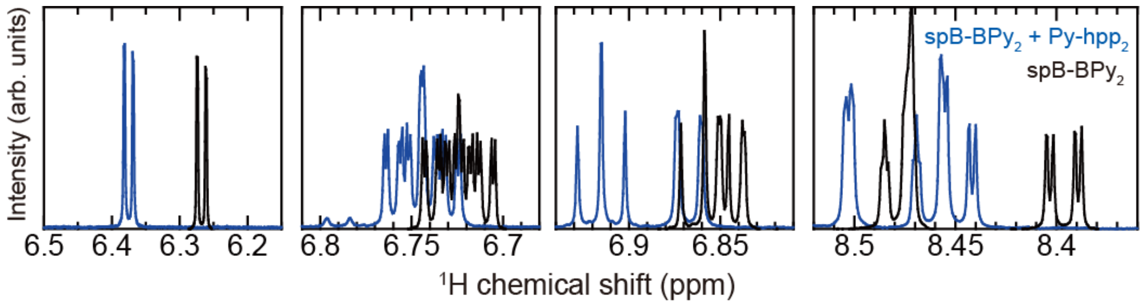

Supplementary Figure 7 | Partial ^{1}H NMR spectra of spB-BPy$_2$ (4.2 mM) upon mixing with Py-hpp$_2$ (91 mM) in benzene-d6. Several shifts were observed by adding Py-hpp$_2$ as in the case of spB-BPy$_2$ mixed with DBN[2]. Thus, it can be concluded that Py-hpp$_2$ forms H-bonds with other organic semiconductors (see also Supplementary Figure 8).

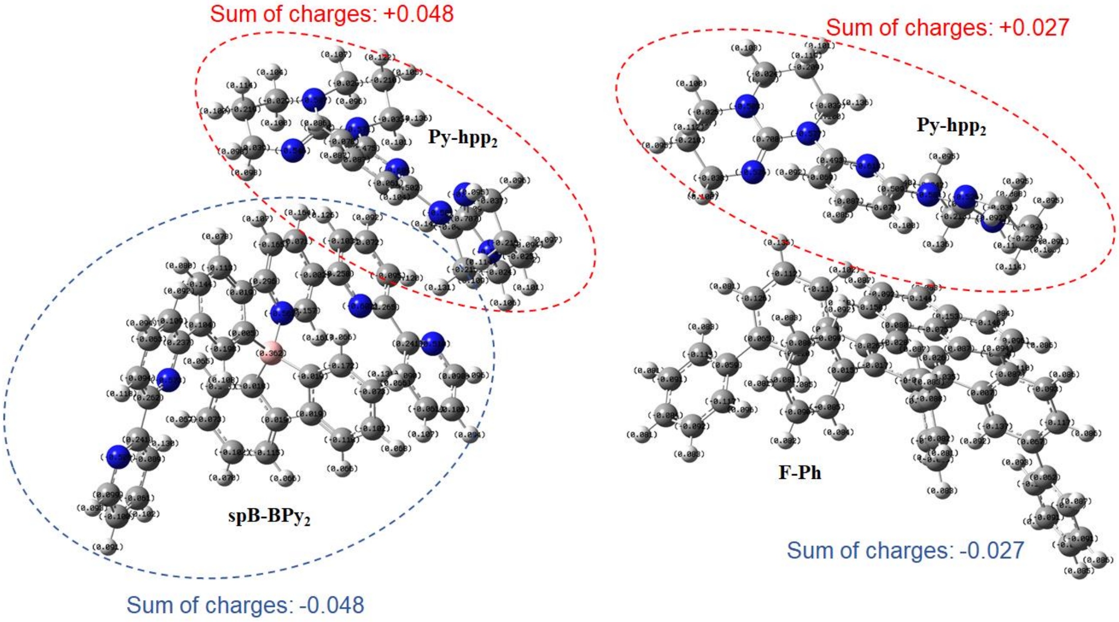

Supplementary Figure 8 | DFT calculation for two-molecule system. Schematic of atomic charge distribution (Mulliken) for (A) spB-BPy$_2$ with Py-hpp$_2$ system and (B) F-Ph with Py-hpp$_2$ system estimated by DFT calculation.

There is polarisation between spB-BPy$_2$ (–δ) and Py-hpp$_2$ (+δ), as in the case of the DFT calculation for spB-BPy$_2$ with DBN[2]. Similar polarisation is obtained between F-Ph (–δ) and Py-hpp$_2$ (+δ). Although the amount of polarisation is similar for DBN and Py-hpp$_2$ in the two-molecule system, the change in WF caused by adding Py-hpp$_2$ is much larger than that upon adding DBN. This difference may be caused by the difference in the number of base units: Py-hpp$_2$ consists of two hpp substituents and one pyridine substituent. It is not clear which substituents are involved in the H-bond/coordination reaction now. In future, we should perform an even more detailed examination of the effects of the base on the change in WF, such as the effects of the number of bases in a single molecule and the kind of substituent.

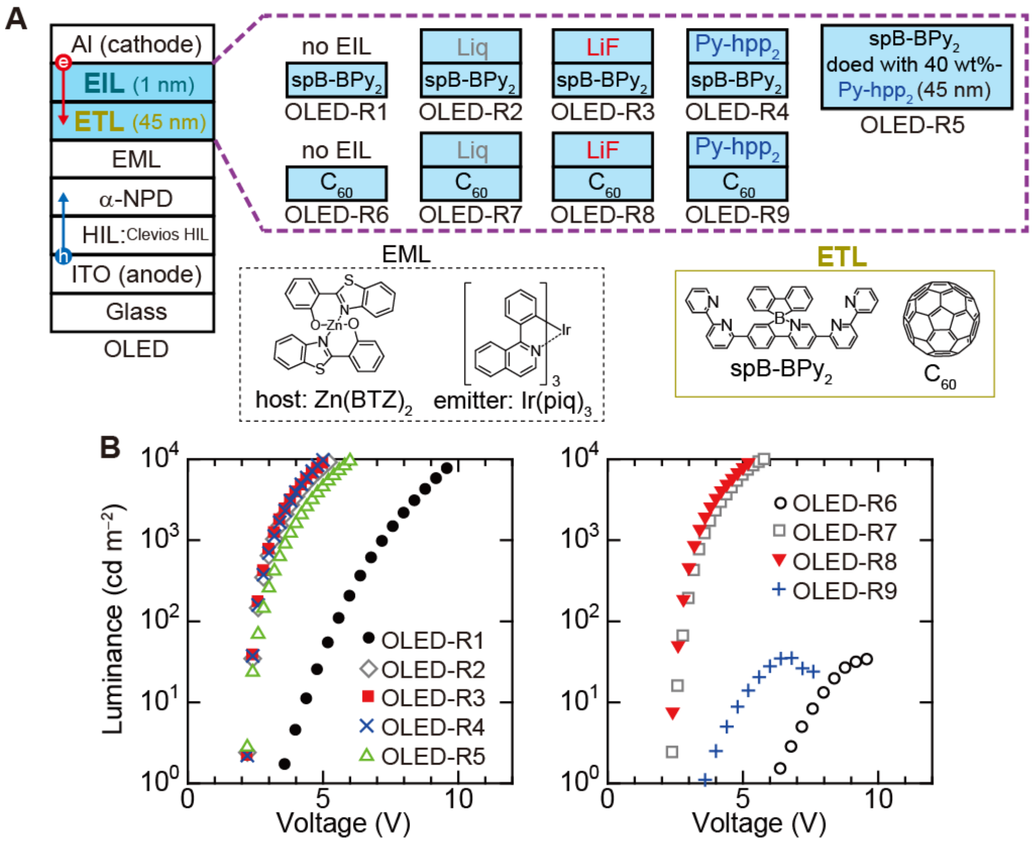

Supplementary Figure 9 | (A) Multilayer structure of a red OLED, chemical structure of the materials used in the OLEDs and schematic illustrations of EIL/ETL combination. (B) Luminance–voltage characteristics of red phosphorescent OLEDs fabricated using various ETL/EIL combinations.

The reason for selecting a red emitter in the OLED with C_{60} is that C_{60} absorbs green/blue emission. The EIL-dependent luminance–voltage characteristics of red OLEDs fabricated using spB-BPy$_2$ as the ETL are similar to those of green OLEDs fabricated using F-Py as the ETL (Fig. 1b). In the case of the red OLEDs with C_{60} as the ETL, on the other hand, the effect of the EIL on the luminance–voltage characteristics is different. Although the operating voltages of the OLEDs with C_{60}/Li compounds are comparable to those of the OLEDs with spB-BPy$_2$/EILs, the operating voltage of the OLED with C_{60}/Py-hpp$_2$ is relatively high. This relatively high operating voltage is likely to originate from the lack of H-bonds. We see from the luminance–voltage characteristics of OLED-R5 that the operating voltage can increase when the Py-hpp$_2$-doped film is thick. A similar increase in the operating voltage was observed when another base was highly doped into the electron-only device[2].

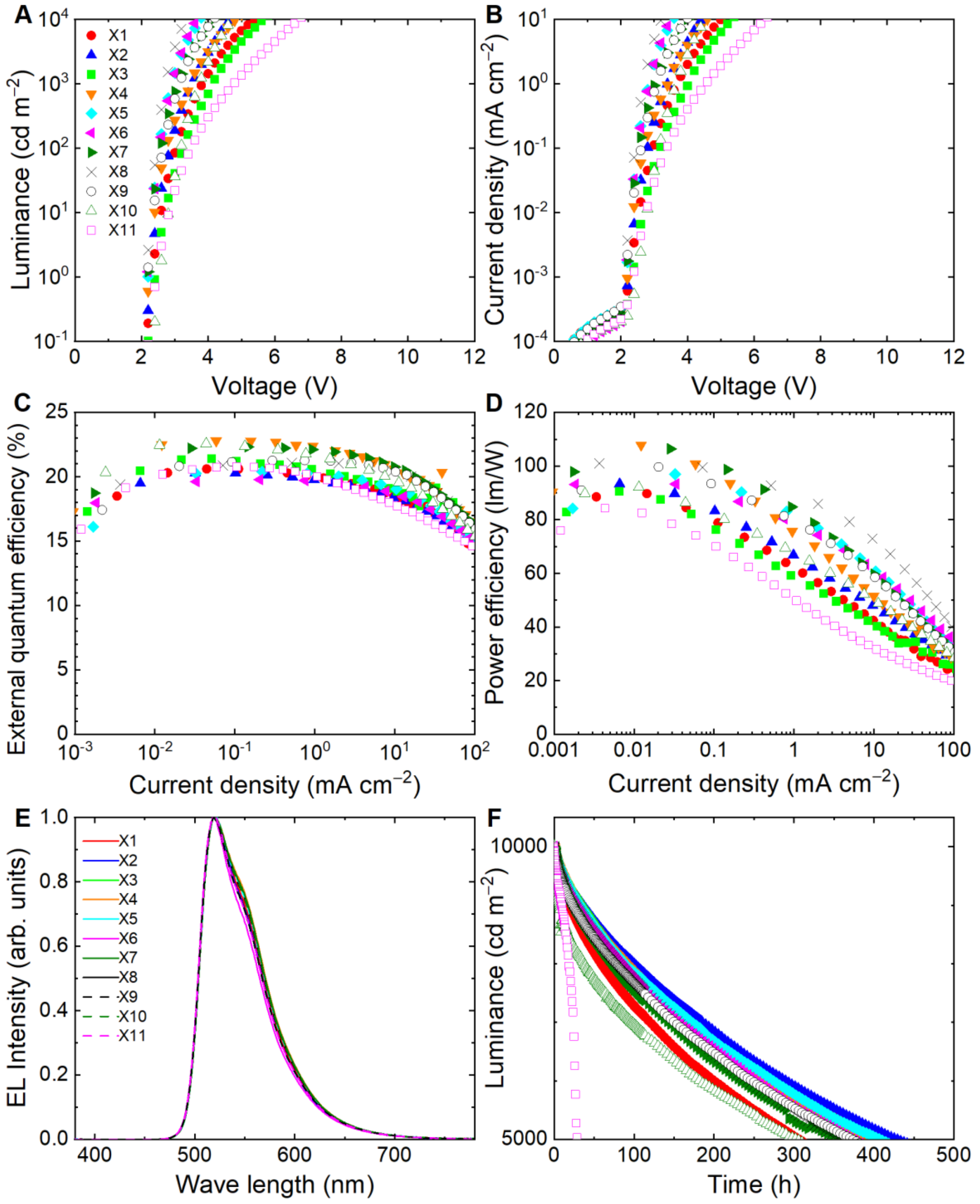

Supplementary Figure 10 | (A) Luminance–voltage and (B) current density–voltage characteristics of OLEDs fabricated using group X as ETL and Liq as EIL. (C) EQE–current density curves of OLEDs. (D) Power efficiency–current density curves of OLEDs. (E) Normalized EL spectra of OLEDs. (F) Luminance–time characteristics of OLEDs under a constant dc with an initial luminance of 10,000 cd m^{-2}.

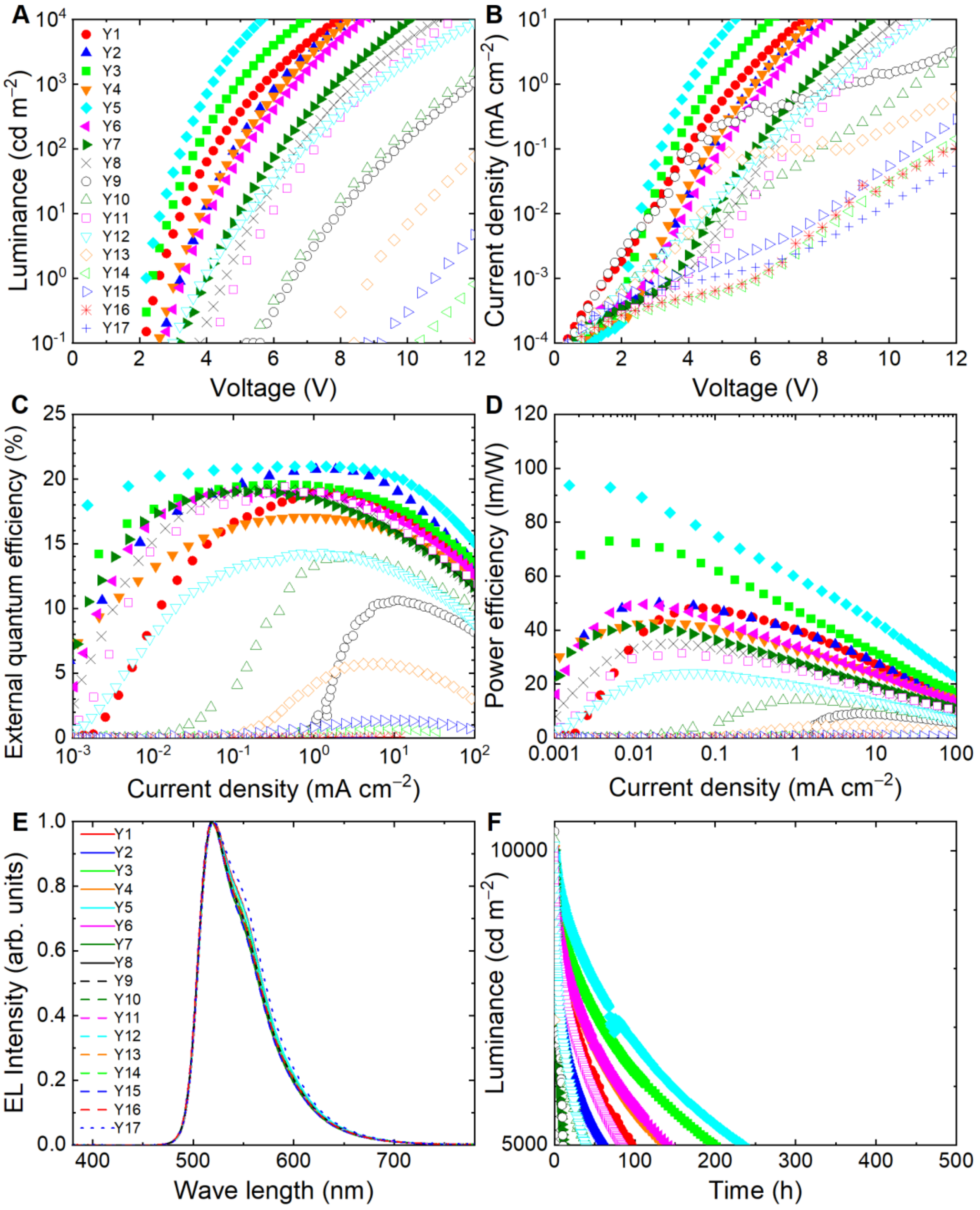

Supplementary Figure 11 | (A) Luminance–voltage and (B) current density–voltage characteristics of OLEDs fabricated using group Y as ETL and Liq as EIL. (C) EQE–current density curves of OLEDs. (D) Power efficiency–current density curves of OLEDs. (E) Normalized EL spectra of OLEDs. (F) Luminance–time characteristics of OLEDs under a constant dc with an initial luminance of 10,000 cd m^{-2}.

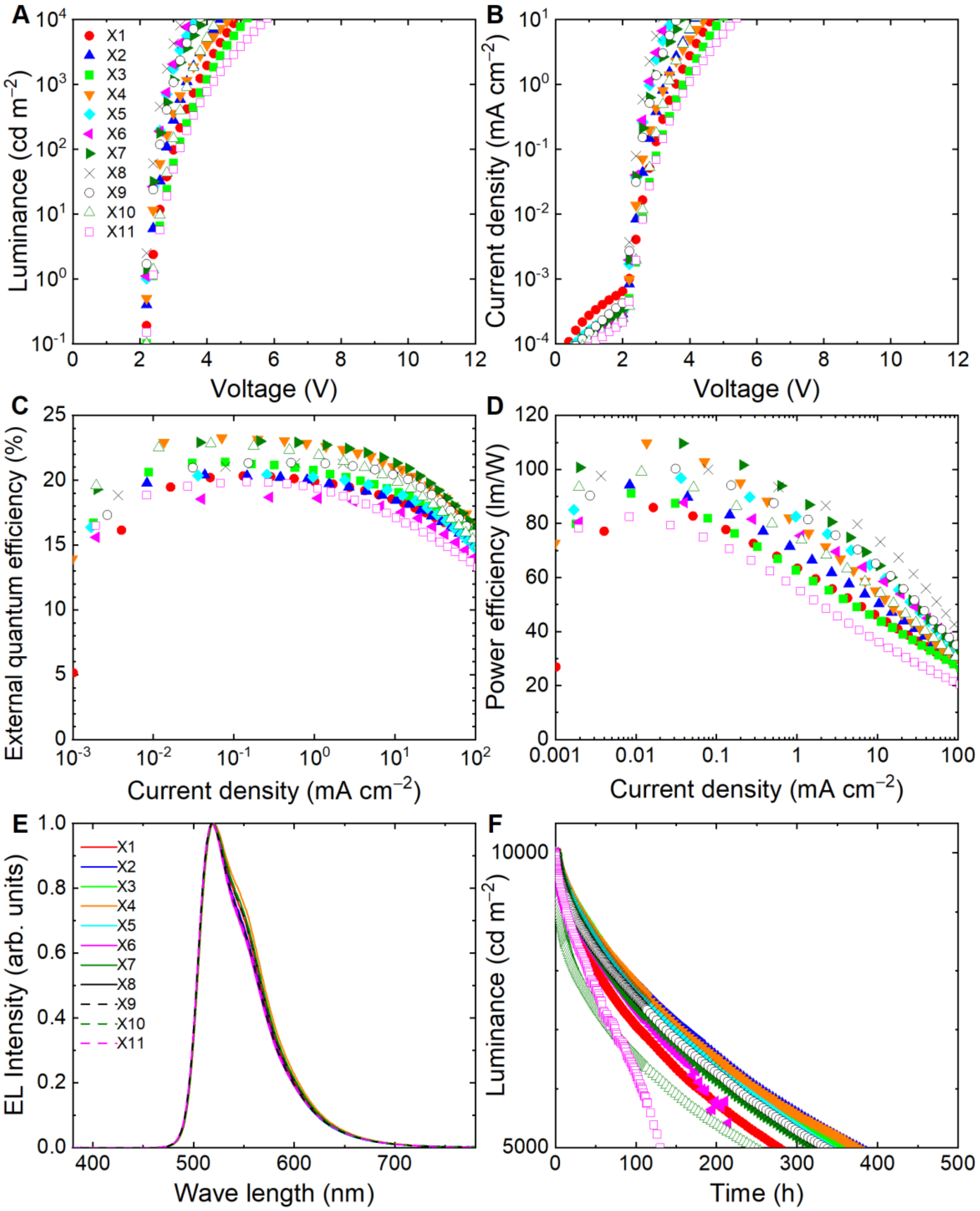

Supplementary Figure 12 | (A) Luminance–voltage and (B) current density–voltage characteristics of OLEDs fabricated using group X as ETL and LiF as EIL. (C) EQE–current density curves of OLEDs. (D) Power efficiency–current density curves of OLEDs. (E) Normalized EL spectra of OLEDs. (F) Luminance–time characteristics of OLEDs under a constant dc with an initial luminance of 10,000 cd m^{-2}.

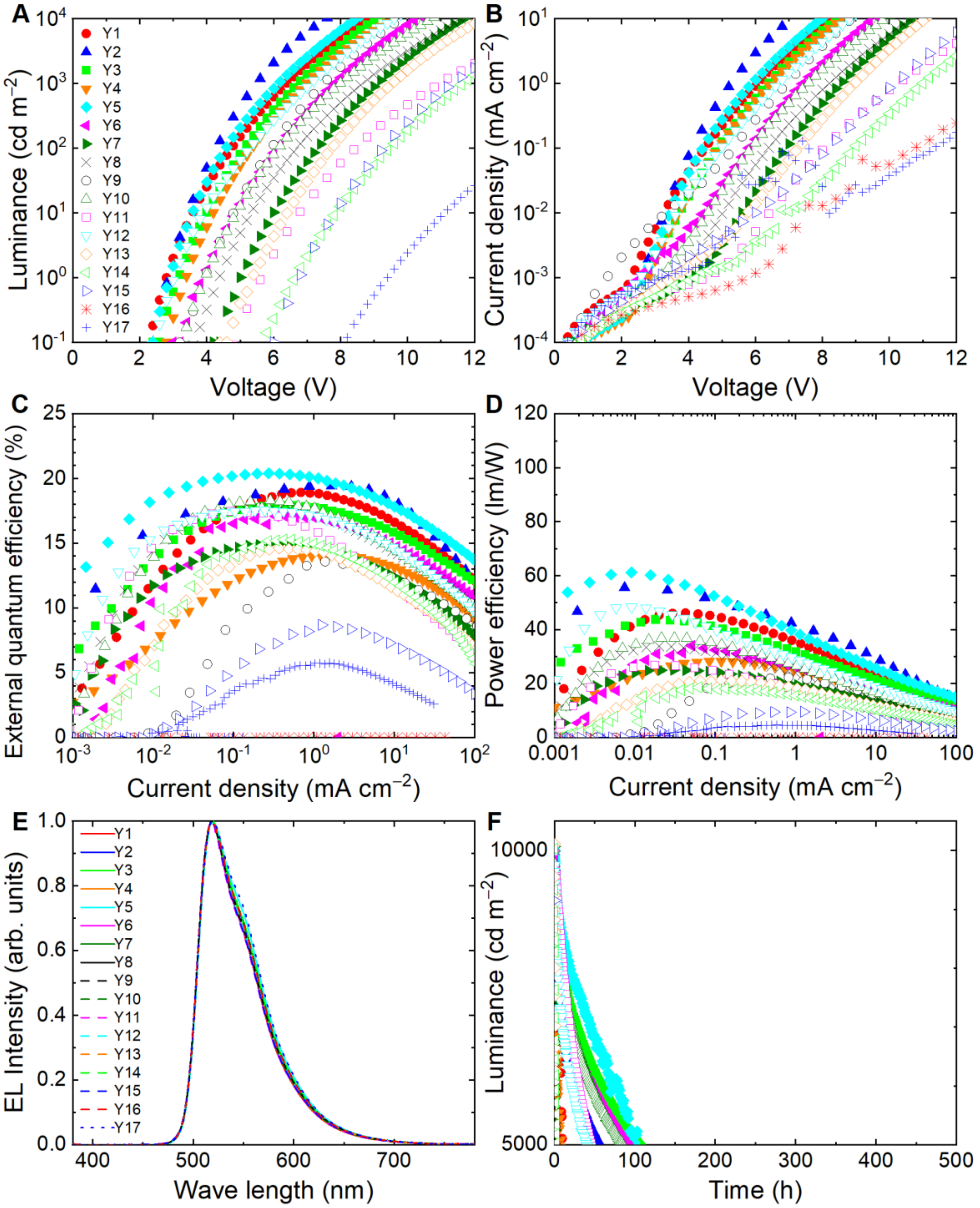

Supplementary Figure 13 | (A) Luminance–voltage and (B) current density–voltage characteristics of OLEDs fabricated using group Y as ETL and LiF as EIL. (C) EQE–current density curves of OLEDs. (D) Power efficiency–current density curves of OLEDs. (E) Normalized EL spectra of OLEDs. (F) Luminance–time characteristics of OLEDs under a constant dc with an initial luminance of 10,000 cd m^{-2}.

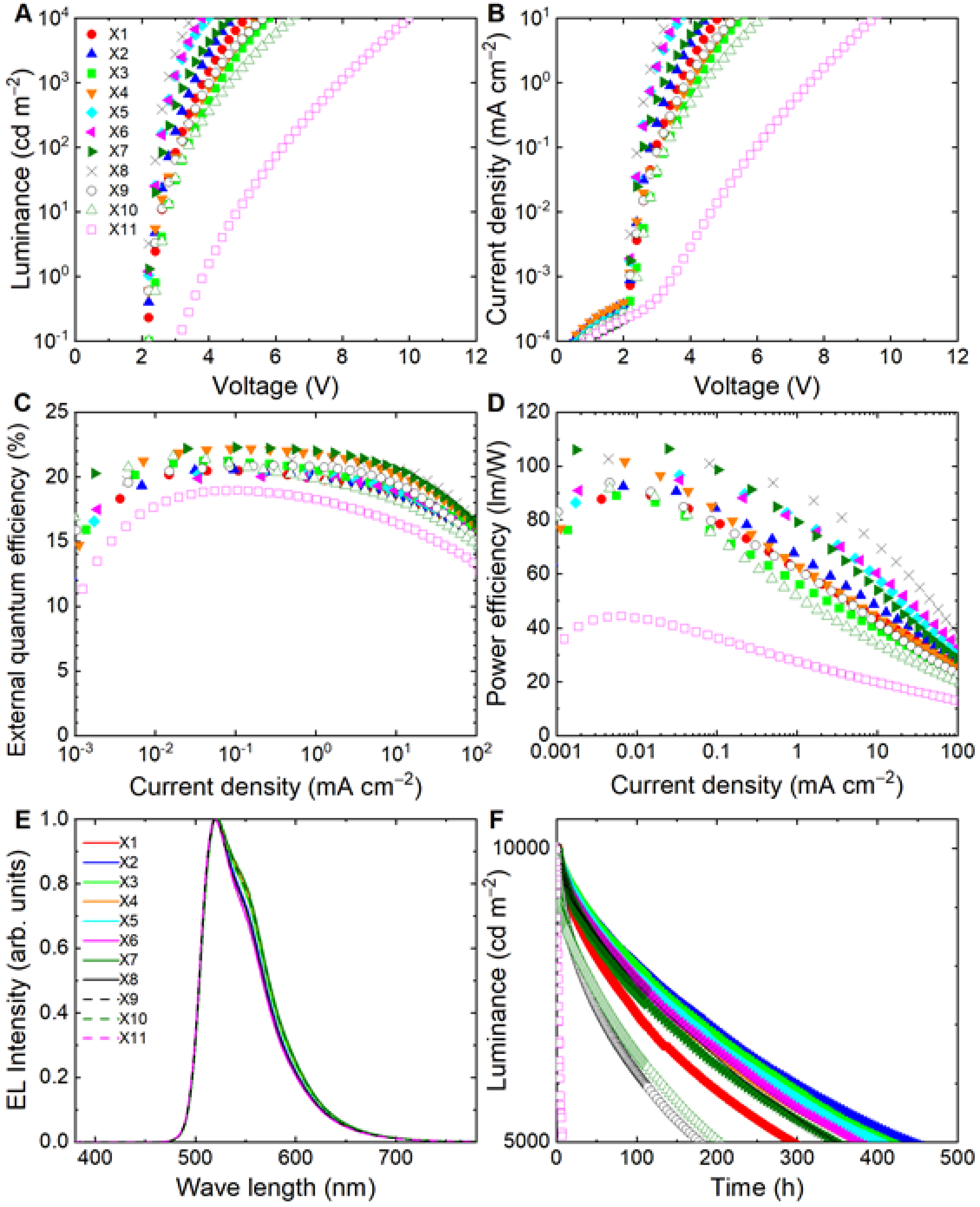

Supplementary Figure 14 | (A) Luminance–voltage and (B) current density–voltage characteristics of OLEDs fabricated using group X as ETL and 1-nm-thick Py-hpp$_2$ as EIL. (C) EQE–current density curves of OLEDs. (D) Power efficiency–current density curves of OLEDs. (E) Normalized EL spectra of OLEDs. (F) Luminance–time characteristics of OLEDs under a constant dc with an initial luminance of 10,000 cd m^{-2}.

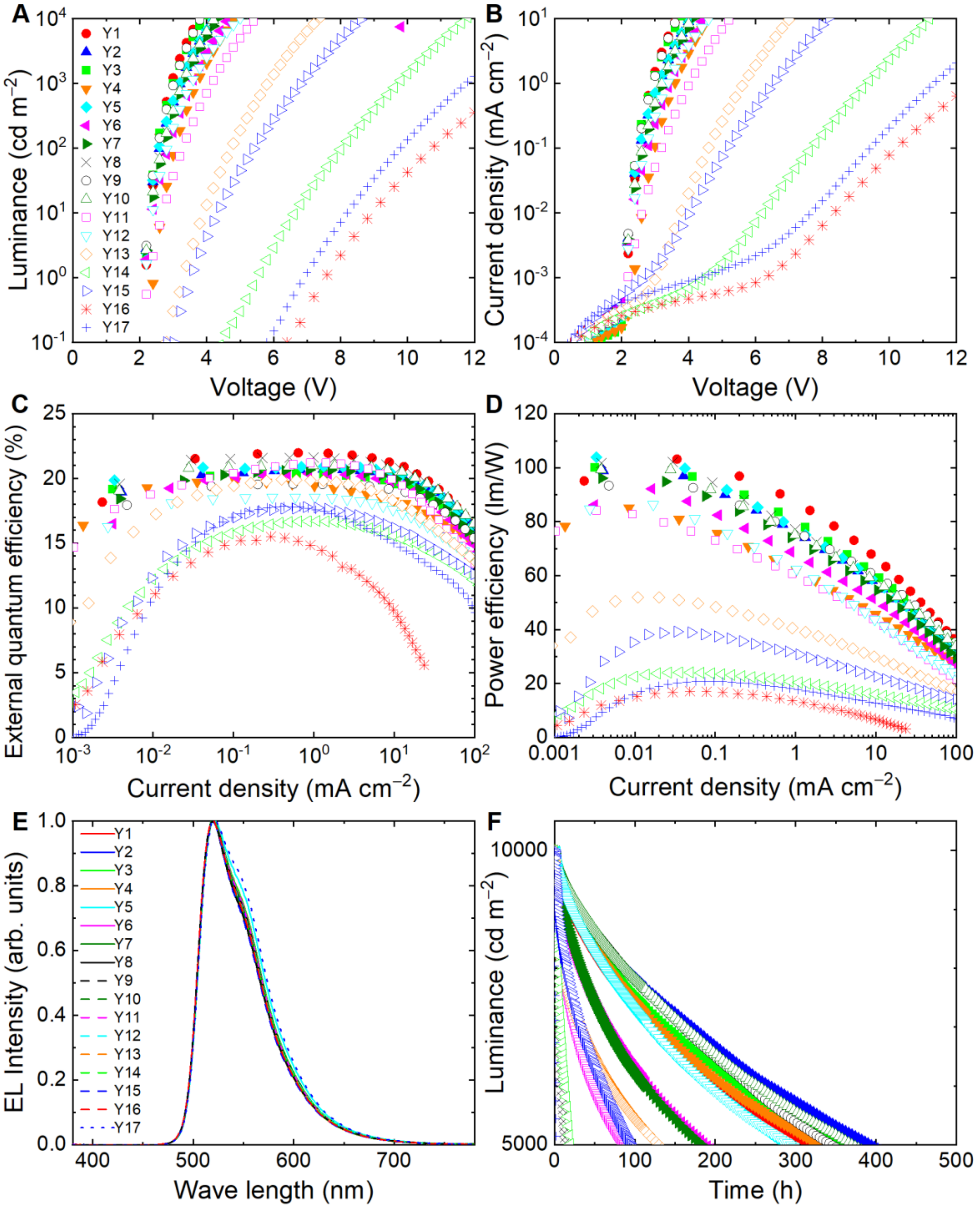

Supplementary Figure 15 | (A) Luminance–voltage and (B) current density–voltage characteristics of OLEDs fabricated using group Y as ETL and 1-nm-thick Py-hpp$_2$ as EIL. (C) EQE–current density curves of OLEDs. (D) Power efficiency–current density curves of OLEDs. (E) Normalized EL spectra of OLEDs. (F) Luminance–time characteristics of OLEDs under a constant dc with an initial luminance of 10,000 cd m^{-2}.

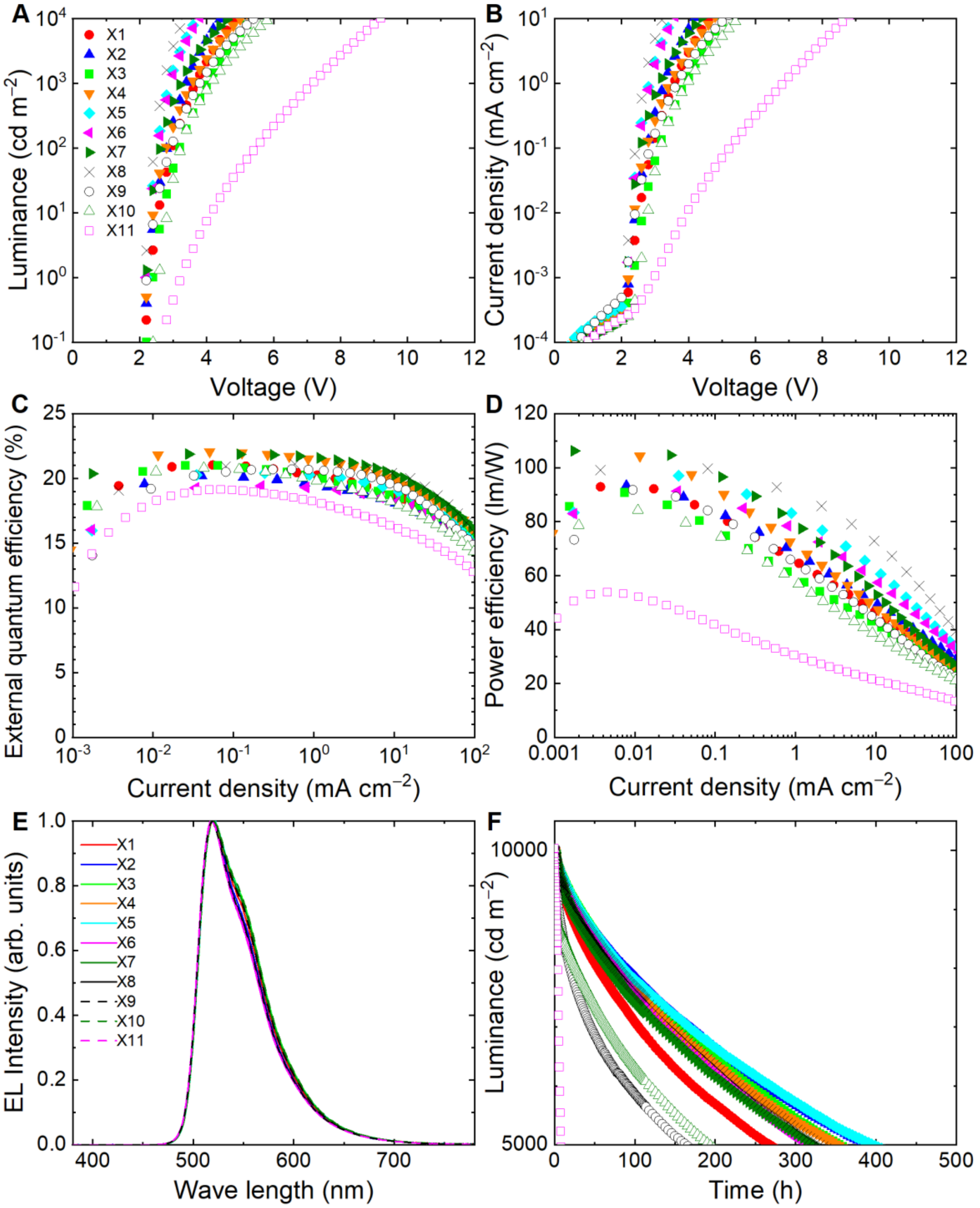

Supplementary Figure 16 | (A) Luminance–voltage and (B) current density–voltage characteristics of OLEDs fabricated using group X as ETL and 5-nm-thick Py-hpp$_2$-doped film as EIL. (C) EQE–current density curves of OLEDs. (D) Power efficiency–current density curves of OLEDs. (E) Normalized EL spectra of OLEDs. (F) Luminance–time characteristics of OLEDs under a constant dc with an initial luminance of 10,000 cd m^{-2}.

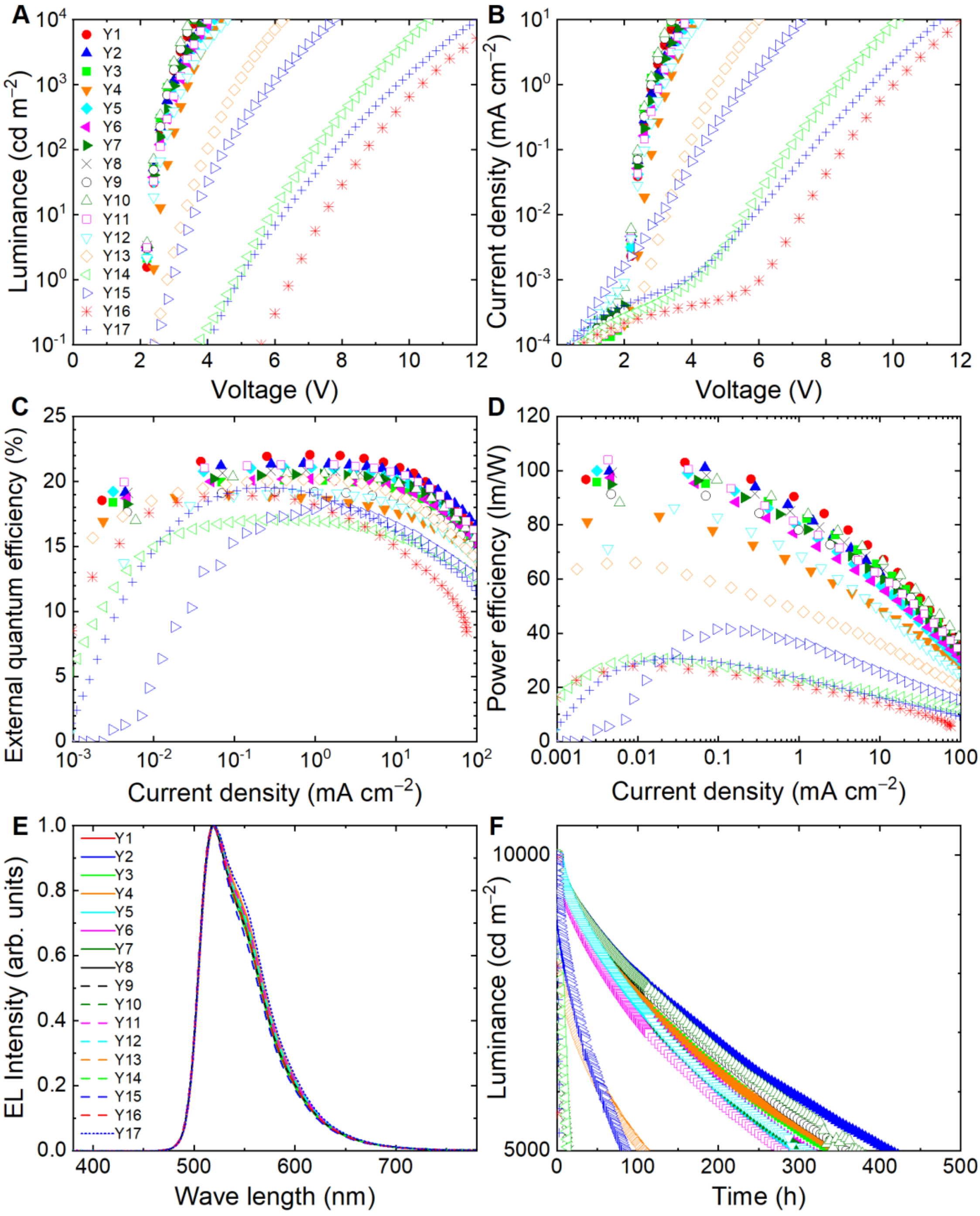

Supplementary Figure 17 | (A) Luminance–voltage and (B) current density–voltage characteristics of OLEDs fabricated using group Y as ETL and 5-nm-thick Py-hpp$_2$-doped film as EIL. (C) EQE–current density curves of OLEDs. (D) Power efficiency–current density curves of OLEDs. (E) Normalized EL spectra of OLEDs. (F) Luminance–time characteristics of OLEDs under a constant dc with an initial luminance of 10,000 cd m^{-2}.

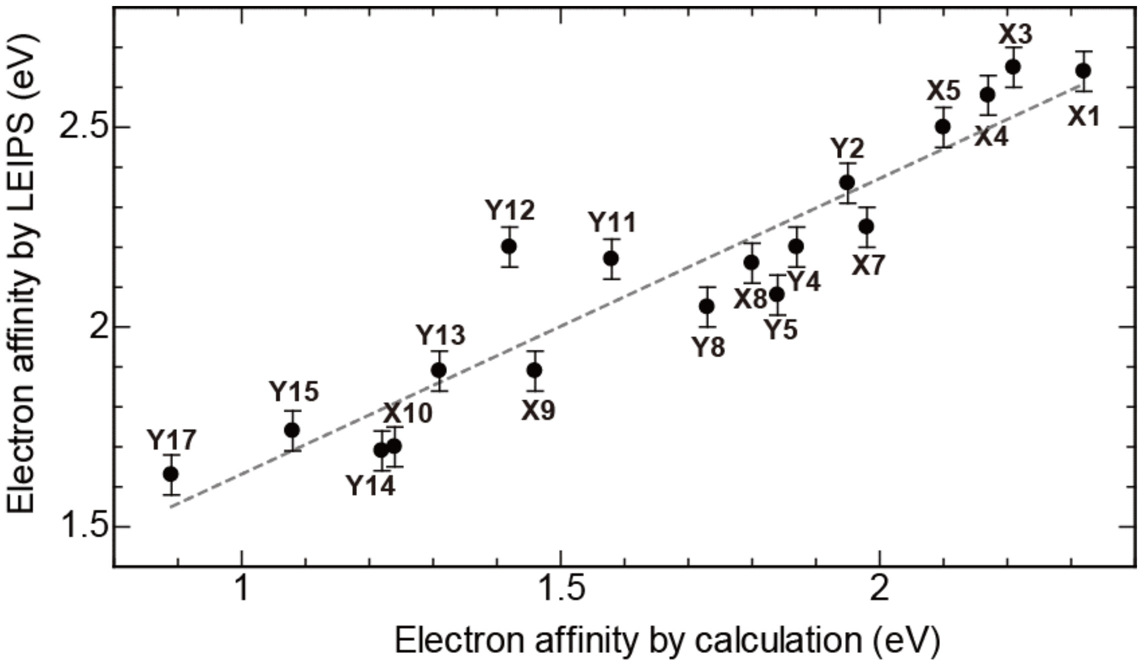

Supplementary Figure 18 | Electron affinity determined by LEIPS compared with calculated values. The strong correlation between EA obtained by LEIPS and EA obtained by calculation was confirmed[12]. Since we were unable to measure the electron mobility of materials, the EA of materials, the electron mobility of which is known, was measured preferentially.

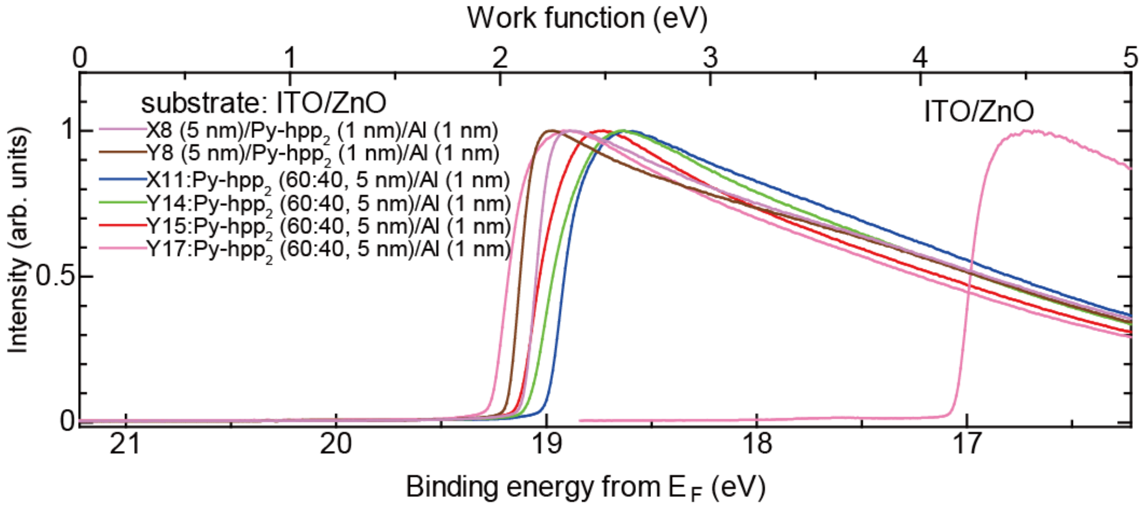

Supplementary Figure 19 | HeI UPS spectra of each film in the secondary region. The work function (WF) around the Al cathode can be seen from the cutoff positions of the UPS spectra.

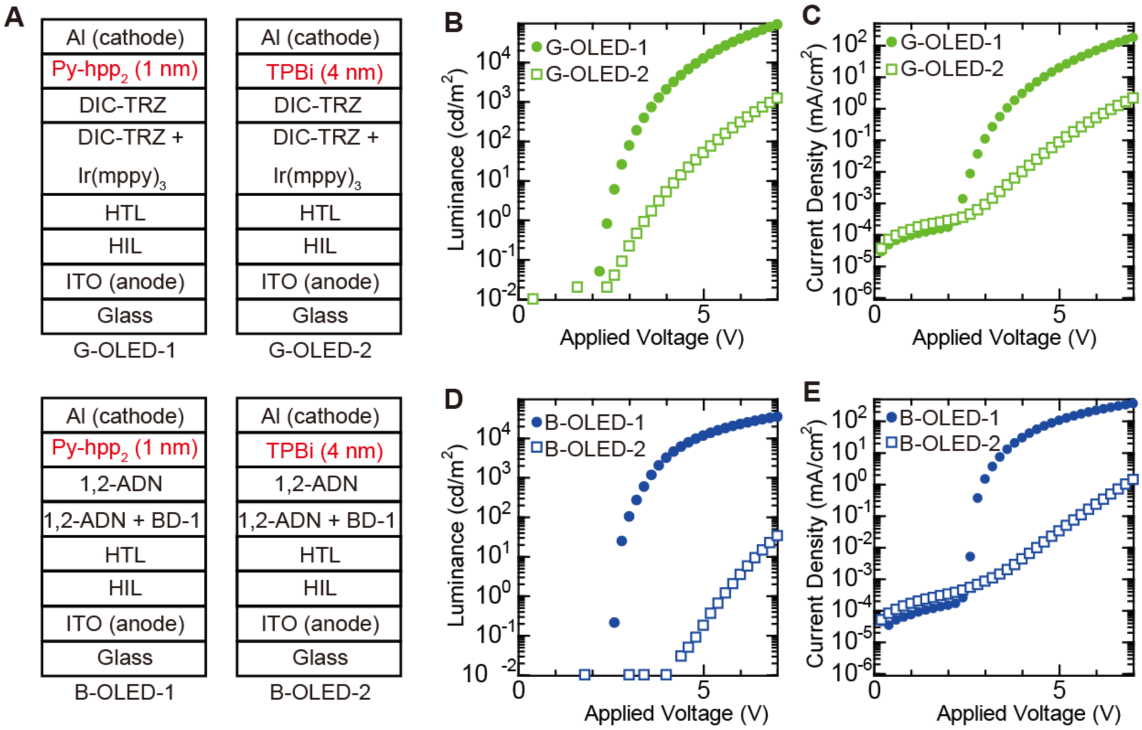

Supplementary Figure 20 | (A) Multilayer structure of green and blue OLEDs. (B, D) Luminance–voltage and (C, E) current density–voltage characteristics of OLEDs fabricated using Py-hpp$_2$ or TPBi as EIL[13].

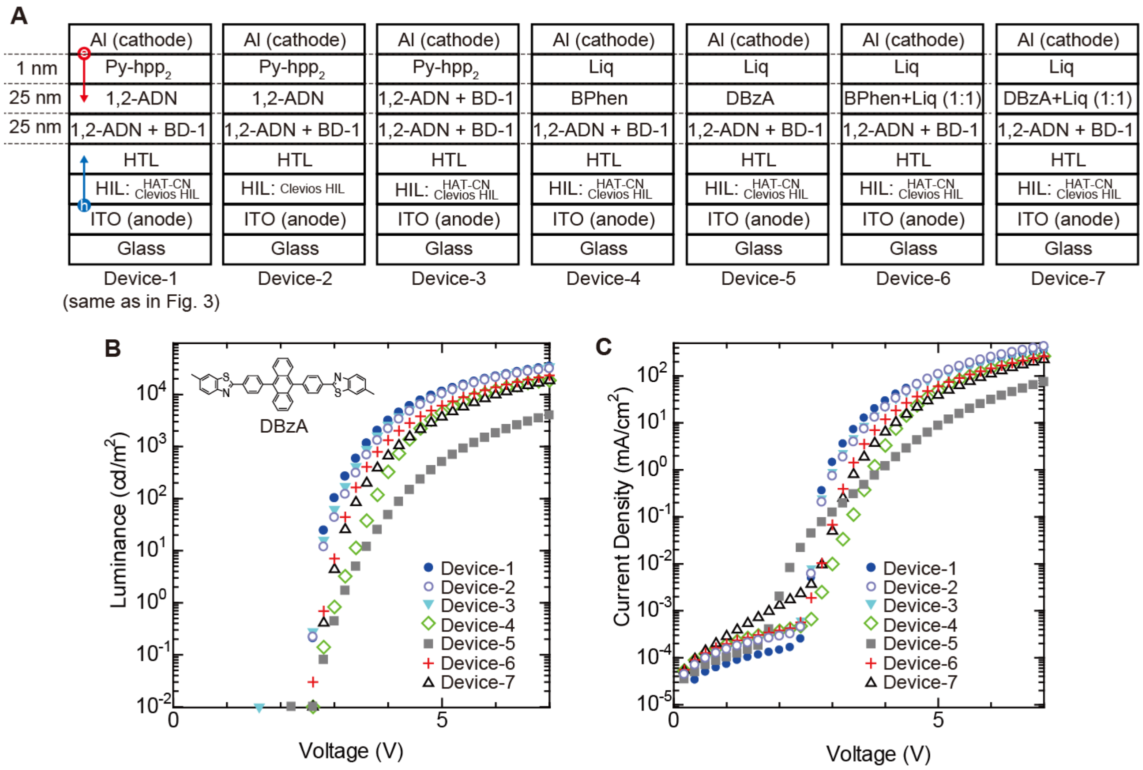

Supplementary Figure 21 | (A) Multilayer structure of blue OLEDs. (B) Luminance–voltage and (C) current density–voltage characteristics of blue OLEDs. Inset: chemical structure of DBzA.

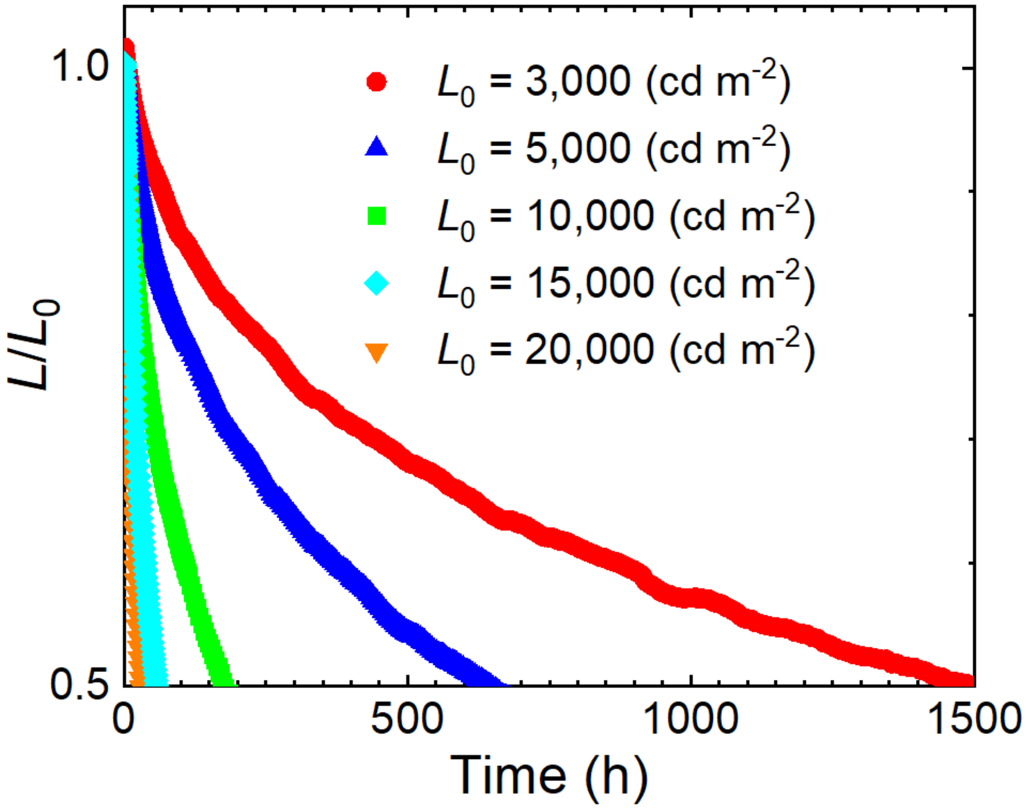

Supplementary Figure 22 | Luminance–time characteristics of a blue OLED under a constant dc with various initial luminance values.

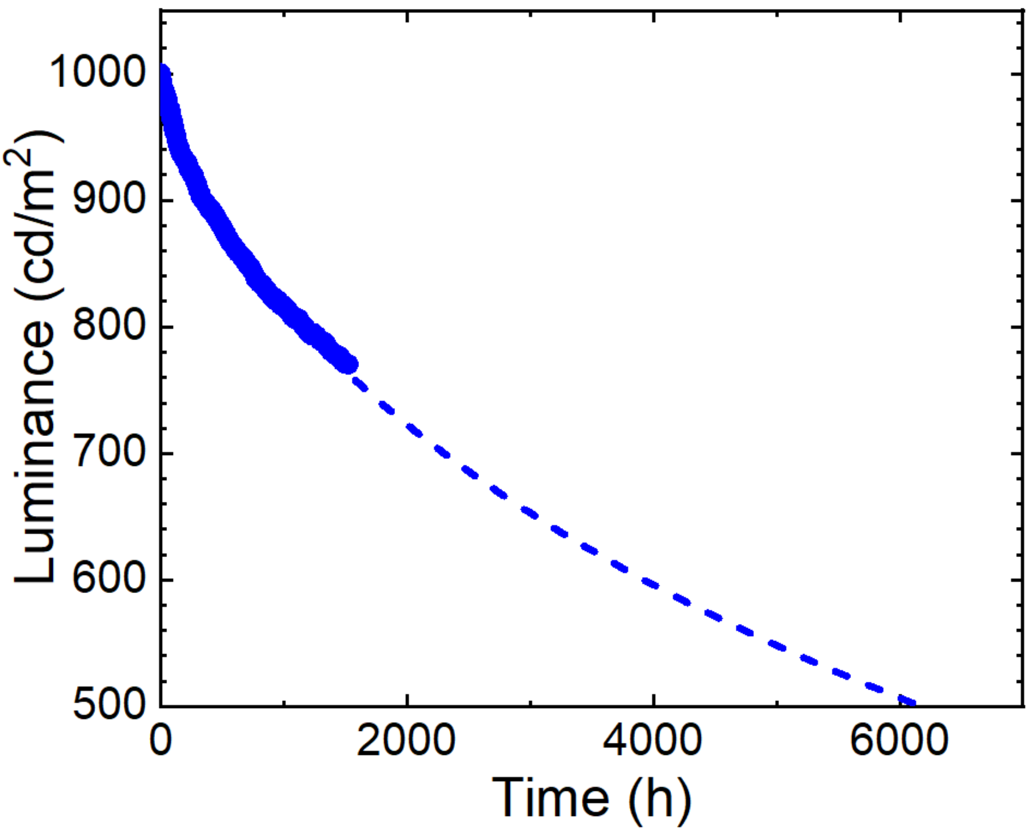

Supplementary Figure 23 | Luminance–time characteristics of a blue OLED under a constant dc with initial luminance of 1,000 cd m^{-2}. LT50 was estimated to be about 6,000 h using the well-known stretched exponential decay function[15].

Supplementary Tables

Supplementary Table 1 | Summary of energies related to EIB in each OLED. Energies of organic thin films before depositing Al are also summarised.

	Cutoff (eV)	WF (eV)	EA (eV)	EIB (eV)
ITO/ZnO/F-Py (5 nm)/Al (1 nm) [related OLED: OLED-1]	18.20	3.02	2.8	0.22
ITO/ZnO/F-Py (5 nm)/Al (3 nm) [OLED-1]	18.16	3.06	2.8	0.26
ITO/ZnO/F-Py (5 nm)/Liq (1 nm)/Al (1 nm) [OLED-2]	18.40	2.82	2.8	~ 0.02
ITO/ZnO/F-Py (5 nm)/Py-hpp$_2$ (1 nm)/Al (1 nm) [OLED-4]	19.11	2.11	*2.16 (*expected EA)	**~ 0 (**expected EIB)
ITO/ZnO/F-Ph (5 nm)/Al (1 nm) [OLED-5]	17.64	3.58	2.05	1.53
ITO/ZnO/F-Ph (5 nm)/Liq (1 nm)/Al (1 nm) [OLED-6]	18.12	3.1	2.05	1.05
ITO/ZnO/F-Ph:Liq (ratio = 60:40, 5 nm)/Al (1 nm) [OLED-6]	18.32	2.9	2.05	0.85
ITO/ZnO/F-Ph (5 nm)/Py-hpp$_2$ (1 nm)/Al (1 nm) [OLED-8]	19.17	2.05	*2.05 (*expected EA)	**~ 0 (**expected EIB)
	Cutoff (eV)	WF (eV)	Onset of HOMO (eV)	IP (eV)
ITO/ZnO/F-Ph (5 nm)	16.78	4.44	1.76	6.20
ITO/ZnO/F-Ph:Py-hpp$_2$ (60:40, 5 nm)	18.53	2.69	3.35	6.04
ITO/ZnO/F-Ph (5 nm)/Py-hpp$_2$ (1 nm)	18.45	2.77	3.35	6.12
ITO/ZnO/F-Ph:LiF (60:40, 5 nm)	18.0	3.22	2.94	6.16
ITO/ZnO/F-Ph:Liq (60:40, 5 nm)	18.20	3.02	3.11	6.13

Cutoff: cutoff position of UPS spectrum shown in Supplementary Figure 3.
WF: work function estimated from cutoff position.
EA: electron affinity measured by LEIPS.
EIB: electron injection barrier from Al to F-Ph or F-Py estimated from WF and EA.
IP: ionisation potential of each film estimated from WF and onset of HOMO.
*expected EA/**expected EIB: The LEIPS results are too complicated to estimate EAs of F-Ph and F-Py in the ITO/ZnO/F-Ph (5 nm)/Py-hpp$_2$ (1 nm) and ITO/ZnO/F-Ph (5 nm)/Py-hpp$_2$ (1 nm)/Al (1 nm) systems. However, from the thermodynamic equilibrium, the EAs of F-Ph and F-Py are likely to be close to their pristine EAs, and the EIBs are expected to be zero.

Supplementary Table 2 | Performances of OLEDs with various EIL/ETL combinations (ETL: group X).

ETL	Calc. (actual) EA of ETM (eV)	Voltage at current density of 1 mA cm^{-2} using various EILs (V)				LT50 from 10,000 cd m^{-2} (h) *[from 1,000 cd m^{-2}]			
		LiF	Liq	1 nm-Py-hpp$_2$	Py-hpp$_2$-doped	LiF	Liq	1 nm-Py-hpp$_2$	Py-hpp$_2$-doped
X1	2.32 (2.64)	3.6	3.7	3.7	3.6	283 [17,900]	316 [19.900]	294 [18,600]	268 [16.900]
X2	2.23	3.3	3.4	3.4	3.3	376 [23,700]	428 [27,000]	441 [27,900]	389 [24,500]
X3	2.21 (2.65)	3.8	4.0	4.2	3.9	357 [22,500]	383 [24,200]	415 [26,200]	355 [22,400]
X4	2.17 (2.58)	3.3	3.4	4.0	3.5	374 [23,600]	399 [25,200]	378 [23,900]	352 [22,200]
X5	2.10 (2.50)	2.8	2.8	2.9	2.8	339 [21,400]	407 [25,700]	402 [25,400]	396 [25,000]
X6	2.10	2.8	2.8	2.9	2.8	218 [13,800]	373 [23,600]	375 [23,700]	315 [19,900]
X7	1.98 (2.25)	2.9	3.0	3.2	3.1	324 [20,500]	355 [22,400]	350 [22,100]	324 [20,400]
X8	1.80 (2.16)	2.6	2.7	2.7	2.7	326 [20,600]	357 [22,500]	348 [22,000]	330 [20,800]
X9	1.56	2.9	3.1	3.9	3.7	336 [21,200]	373 [23,500]	179 [11,300]	164 [10,300]
X10	1.46 (1.89)	3.4	3.7	4.4	4.0	247 [15,600]	303 [19,100]	203 [12,800]	193 [12,100]
X11	1.24 (1.70)	3.9	4.5	7.6	6.8	130 [8,200]	29 [1,800]	7 [500]	8 [500]

*The LT50s from 1,000 cd m^{-2} were extrapolated by data plots and the equation.
$L_0^n t_{1/2} = const,$
where n is the acceleration coefficient, L_0 is the initial luminance and $t_{1/2}$ is the half-lifetime[14-16]. The acceleration coefficient n was estimated to be 1.8 from LT50s of OLED with X5/Py-hpp$_2$-doped-EIL with different initial luminance.

Supplementary Table 3 | Performances of OLEDs with various EIL/ETL combinations (ETL: group Y).

ETL	Calc. (actual) EA of ETM (eV)	Voltage at current density of 1 mA cm^{-2} using various EILs (V)				LT50 from 10,000 cd m^{-2} (h) *[from 1,000 cd m^{-2}]			
		LiF	Liq	1 nm-Py-hpp$_2$	Py-hpp$_2$-doped	LiF	Liq	1 nm-Py-hpp$_2$	Py-hpp$_2$-doped
Y1	2.03	6.1	5.4	2.9	2.8	10 [600]	94 [5,900]	319 [20,100]	294 [18,500]
Y2	1.95 (2.36)	5.3	5.9	3.1	2.9	57 [3,600]	63 [3,900]	393 [24,800]	413 [26,100]
Y3	1.94	6.4	4.7	2.9	2.7	105 [6,600]	199 [12,600]	351 [22,200]	343 [21,600]
Y4	1.87 (2.20)	6.4	6.0	3.6	3.3	7 [400]	135 [8,500]	329 [20,800]	350 [22,000]
Y5	1.84 (2.08)	5.9	4.0	3.1	3.0	100 [6,300]	235 [14,800]	290 [18,300]	288 [18,200]
Y6	1.79	7.4	6.4	3.4	3.0	90 [5,700]	141 [8,900]	188 [11,900]	311 [19,600]
Y7	1.74	8.5	7.5	3.2	2.9	1 [40]	11 [700]	183 [11,500]	309 [19,500]
Y8	1.73 (2.05)	8.2	8.1	3.2	2.8	3 [200]	2 [100]	14 [900]	14 [900]
Y9	1.66	6.9	8.7	2.9	2.8	2 [100]	11 [700]	345 [21,800]	359 [22,600]
Y10	1.65	7.6	10.8	3.2	2.7	85 [5,400]	39 [2,500]	371 [23,400]	373 [23,500]
Y11	1.58 (2.17)	10.3	8.6	4.0	3.0	46 [2,900]	84 [5,300]	86 [5,400]	279 [17,600]
Y12	1.42 (2.20)	6.7	8.3	3.4	3.2	40 [2,500]	38 [2,400]	294 [18,500]	294 [18,500]
Y13	1.31 (1.89)	9.0	12.7	5.6	4.7	1 [80]	0 [1]	129 [8,200]	108 [6,800]
Y14	1.22 (1.69)	11.0	14.8	9.3	8.4	2 [100]	–	19 [1,200]	14 [900]
Y15	1.08 (1.74)	10.1	13.6	6.5	5.6	0 [2]	–	95 [6,000]	84 [5,300]
Y16	0.93	14.3	15.7	12.5	10.0	–	–	–	0 [5]
Y17	0.89 (1.63)	14.3	17.0	11.3	9.3	–	–	2 [100]	3 [200]

*The LT50s from 1,000 cd m^{-2} were extrapolated by data plots and the equation.

$$L_0^n t_{1/2} = const,$$

where n is the acceleration coefficient, L_0 is the initial luminance and $t_{1/2}$ is the half-lifetime[14-16]. The acceleration coefficient n was estimated to be 1.8 from LT50s of OLED with X5/Py-hpp$_2$-doped-EIL with different initial luminance.

Supplementary Table 4 | Summary of the sublimation temperature of each material when the evaporation rate was about 0.04 nm/s. The organic deposition cell (KOD-Cell, KITANO SEIKI CO., LTD.) was used for evaporation.

Material	Temperature (degree)		Material	Temperature (degree)
α-NPD	~236		Y1	~277
4DBFP3Q	~220		Y2	~258
HTM-081	~212		Y3	~226
1,2-ADN (host in blue OLEDs)	~173		Y4 (host in green OLEDs)	~205
Py-hpp$_2$	~154 (rate: ~ 0.027 nm/s)		Y5	~231
HAT-CN	~251		Y6	~211
Ir(mppy)$_3$	~205 (rate: ~ 0.0012 nm/s)		Y7	~205
BD-1	~250 (rate: ~ 0.0012 nm/s)		Y8 (F-Ph)	~238
X1	~231		Y9	~298
X2	~250		Y10	~337
X3	~229		Y11	~171
X4	~304		Y12	~313
X5	~278		Y13	~231
X6	~251		Y14	~244
X7	~273		Y15	~239
X8 (F-Py)	~249		Y16	~218
X9	~333		Y17	~315
X10	~175			
X11	~255			

Supplementary References

1. Iwasaki, Y., Fukagawa, H. & Shimizu, T. Effect of host moieties on the phosphorescent spectrum of green platinum complex. *Molecules* **24**, 21749–21755 (2019).
2. Fukagawa, H. et al. Universal strategy for efficient electron injection into organic semiconductors utilizing hydrogen bonds. *Adv. Mater.* **31**, e1904201 (2019).
3. Su, S. J., Sasabe, H., Pu, Y. J., Nakayama, K. & Kido, J. Tuning energy levels of electron-transport materials by nitrogen orientation for electrophosphorescent devices with an 'ideal' operating voltage. *Adv. Mater.* **22**, 3311–3316 (2010).
4. Hirata, S. et al. Highly efficient blue electroluminescence based on thermally activated delayed fluorescence. *Nat. Mater.* **14**, 330–336 (2015).
5. Zhang, D., Duan, L., Zhang, D., Qiu, Y. Towards ideal electrophosphorescent devices with low dopant concentrations: the key role of triplet up-conversion. *J. Mater. Chem. C* **2**, 8983–8989 (2014).
6. Hashimoto, S. et al. Triplet-energy control of polycyclic aromatic hydrocarbons by BN replacement: Development of ambipolar host materials for phosphorescent organic light-emitting diodes. *Chem. Mater.* **26**, 6265–6271 (2014).
7. Kaida, H., Satoh, T., Nishii, Y., Hirano, K. & Miura, M. Synthesis of benzobis- and benzotrisbenzofurans by palladium-catalyzed multiple intramolecular C–H/C–H coupling. *Chem. Lett.* **45**, 1069–1071 (2016).
8. Park, S., Yi, Y., Cho S. W. & Lee, H. Work function reduction using 8-hydroxyquinolinolato-lithium for efficient inverted devices. *Chem. Phys. Lett.* **652**, 102–105 (2016).
9. Isshiki, Y., Fujii, S., Nishino, T. & Kiguchi, M. Fluctuation in interface and electronic structure of single-molecule junctions investigated by current versus bias voltage characteristics. *J. Am. Chem. Soc.* **140**, 3760–3767 (2018).
10. Bin, Z. et al. Making silver a stronger n-dopant than cesium via in situ coordination reaction for organic electronics. *Nat. Commun.* **10**, 866 (2019).
11. Fukagawa, H. et al. Understanding coordination reaction for producing stable electrode with various low work functions. *Nat. Commun.* **11**, 3700 (2020).
12. Yoshida, H. & Yoshizaki, K. Electron affinities of organic materials used for organic light-emitting diodes: A low-energy inverse photoemission study. *Org. Electron.* **20**, 24–30 (2015).
13. Kotadiya, N. B., Blom, P. W. M. & Wetzelaer, G.-J. A. H. Efficient and stable single-layer organic light-emitting diodes based on thermally activated delayed fluorescence. *Nat. Photon.* **13**, 765–769 (2019).

14. Zhang, Y., Lee, J. & Forrest, S. R. Tenfold increase in the lifetime of blue phosphorescent organic light-emitting diodes. *Nat. Commun.* **5**, 5008 (2014).
15. Féry, C., Racine, B., Vaufrey, D. Doyeux, H. & Cinà, S. Physical mechanism responsible for the stretched exponential decay behavior of aging organic light-emitting diodes. *Appl. Phys. Lett.* **87**, 213502 (2005).
16. Orselli, E., Maunoury, J., Bascour, D., & Catinat, J.-P. Orange phosphorescent organic light-emitting diodes with high operational stability. *Org. Electron.* **13**, 1506–1510 (2012).
